# Discovery of a heavy silicon isotope mantle reservoir

**DOI:** 10.1093/nsr/nwaf410

**Published:** 2025-09-24

**Authors:** Mao-Rui Liu, Jun Wang, Ze-Xian Cui, Gang-Jian Wei, Qing Yang, Yi-Gang Xu, Andrew C Kerr, Derek Wyman, Jiang-Hao Bai, Guan-Hong Zhu, Lin Ma, Lu-Lu Hao, Jin-Sheng Zhou, Jing-Jing Fan, Tong-Yu Huang, Miao-Yan Zhang, Qiang Wang

**Affiliations:** State Key Laboratory of Deep Earth Processes and Resources, Guangzhou Institute of Geochemistry, Chinese Academy of Sciences, Guangzhou 510640, China; College of Earth and Planetary Sciences, University of Chinese Academy of Sciences, Beijing 100049, China; State Key Laboratory of Deep Earth Processes and Resources, Guangzhou Institute of Geochemistry, Chinese Academy of Sciences, Guangzhou 510640, China; State Key Laboratory of Deep Earth Processes and Resources, Guangzhou Institute of Geochemistry, Chinese Academy of Sciences, Guangzhou 510640, China; State Key Laboratory of Deep Earth Processes and Resources, Guangzhou Institute of Geochemistry, Chinese Academy of Sciences, Guangzhou 510640, China; College of Earth and Planetary Sciences, University of Chinese Academy of Sciences, Beijing 100049, China; State Key Laboratory of Deep Earth Processes and Resources, Guangzhou Institute of Geochemistry, Chinese Academy of Sciences, Guangzhou 510640, China; State Key Laboratory of Deep Earth Processes and Resources, Guangzhou Institute of Geochemistry, Chinese Academy of Sciences, Guangzhou 510640, China; College of Earth and Planetary Sciences, University of Chinese Academy of Sciences, Beijing 100049, China; School of Earth and Environmental Sciences, Cardiff University, Cardiff CF10 3AT, UK; School of Geosciences, The University of Sydney, Sydney 2006, Australia; State Key Laboratory of Deep Earth Processes and Resources, Guangzhou Institute of Geochemistry, Chinese Academy of Sciences, Guangzhou 510640, China; State Key Laboratory of Deep Earth Processes and Resources, Guangzhou Institute of Geochemistry, Chinese Academy of Sciences, Guangzhou 510640, China; State Key Laboratory of Deep Earth Processes and Resources, Guangzhou Institute of Geochemistry, Chinese Academy of Sciences, Guangzhou 510640, China; State Key Laboratory of Deep Earth Processes and Resources, Guangzhou Institute of Geochemistry, Chinese Academy of Sciences, Guangzhou 510640, China; State Key Laboratory of Deep Earth Processes and Resources, Guangzhou Institute of Geochemistry, Chinese Academy of Sciences, Guangzhou 510640, China; State Key Laboratory of Deep Earth Processes and Resources, Guangzhou Institute of Geochemistry, Chinese Academy of Sciences, Guangzhou 510640, China; State Key Laboratory of Deep Earth Processes and Resources, Guangzhou Institute of Geochemistry, Chinese Academy of Sciences, Guangzhou 510640, China; State Key Laboratory of Deep Earth Processes and Resources, Guangzhou Institute of Geochemistry, Chinese Academy of Sciences, Guangzhou 510640, China; State Key Laboratory of Deep Earth Processes and Resources, Guangzhou Institute of Geochemistry, Chinese Academy of Sciences, Guangzhou 510640, China; College of Earth and Planetary Sciences, University of Chinese Academy of Sciences, Beijing 100049, China

**Keywords:** mantle heterogeneity, Si isotope, lamproites, silicate melt metasomatism, slab melts

## Abstract

Silicon cycling between Earth’s reservoirs provides critical insights into how the Earth operates. While average crust and the bulk silicate Earth (BSE) share similar silicon isotope (δ^30^Si) compositions, some mantle-derived magmas exhibit lower δ^30^Si values than the BSE, implying the existence of an unidentified mantle reservoir with complementary higher δ^30^Si values. We present silicon isotope data from Cenozoic lamproites and their hosted mantle pyroxenite xenoliths from the Himalaya–Tibet orogen. These mantle-derived rocks have higher δ^30^Si values than the BSE, which resulted from reaction between mantle peridotite and ^30^Si-rich silicate melts from subducted Indian continental crust. Our results demonstrate that slab melting can produce high-δ^30^Si melts and complementary low-δ^30^Si residues. These products are unevenly distributed in the mantle, with high-δ^30^Si melts stored as metasomatic veins in the lithospheric mantle while low-δ^30^Si residues are recycled into the deep mantle. This study provides evidence that mantle metasomatism by high-δ^30^Si slab melts creates heavy silicon reservoirs in the lithospheric mantle above continental slabs or cool mantle wedges above oceanic slabs.

## Teaser

Si isotope data from postcollisional lamproites and associated mantle pyroxenite xenoliths from the Himalayan-Tibet orogen show a distinctive heavy Si isotopic signature, possibly linked to mantle metasomatism induced by the subducted continetal slab-derived melts.

## INTRODUCTION

As Earth’s third most abundant element, the distribution and cycling of silicon across various planetary reservoirs provide critical insights into Earth’s internal differentiation and surface environmental evolution [1,2]. For example, the global cycles of silicon (Si) and carbon (C) are thought to be intrinsically linked, as silicate minerals near the Earth’s surface undergo chemical weathering, which regulates atmospheric CO_2_ levels and drives climate stabilization or abrupt transitions [3–5]. The evolutionary radiation of siliceous organisms led to a decrease in dissolved marine Si levels, thus changing the mode of marine C–Si cycles throughout deep time [6]. Earth is unique in the solar system for its compositionally evolved continental crust [7]. The transformation of the original mafic (silica-poor) crust to the present more felsic (silica-rich) crust is closely associated with the shallow migration and deep cycling of silicon [8–10]. Therefore, a comprehensive understanding of the silicon cycle between Earth’s surface and deep reservoirs throughout geological time is essential for elucidating the co-evolution of life and habitability on our planet.

Silicon has three stable isotopes (^28^Si: 92.23%; ^29^Si: 4.68%; and ^30^Si: 3.09%) and its isotopes are widely utilized as a proxy for reconstructing paleoclimate and paleoproductivity [11,12]. Significant silicon isotopic fractionation has been documented during diagenesis [13], biological processes [14], chemical weathering [15–17] and magmatic differentiation [18]. However, the successful application of silicon isotopes as proxies for past and present geochemical cycles hinges on a comprehensive understanding of δ^30^Si values across geological reservoirs [19]. Notably, compared to the well-constrained silicon isotope budget of Earth’s surface, the silicon isotopic composition of the mantle remains poorly characterized.

**Figure 1.**
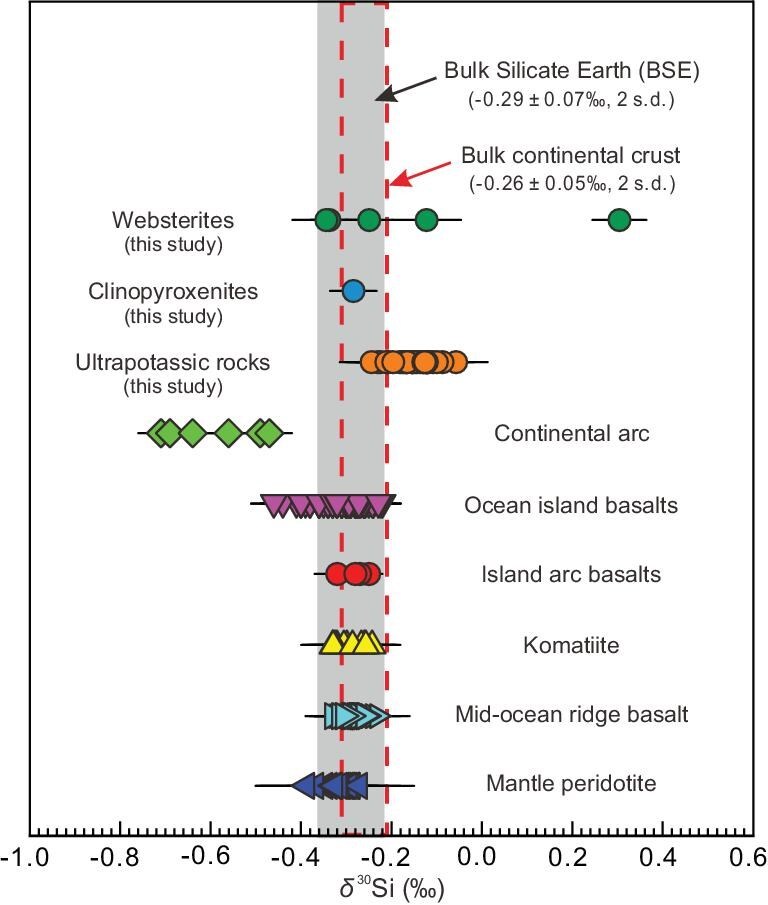
Global compilation of silicon isotopic compositions for mantle rocks and their derived magmas. The shaded box represents the estimate for the Si isotopic composition of the BSE [22]. The bulk composition of continental crust (red dashed line box) is derived from the silicon isotopic compositions for the upper, middle and lower crust as estimated by Savage *et al.* [39] and Savage *et al.* [40], and subsequently mixed in the proportions determined by the global compilation of Rudnick and Gao [71]: 31.7% for the upper crust, 29.6% for the middle crust and 38.8% for the lower crust. Notably, the silicon isotopic composition of the bulk continental crust closely resembles that of the BSE. Data sources: ultramafic xenoliths [24,72], mid-ocean ridge basalt [24,29,72], komatiite [23,30], island arc basalts [24], continental arc [25], ocean island basalts [26,72].

**Figure 2.**
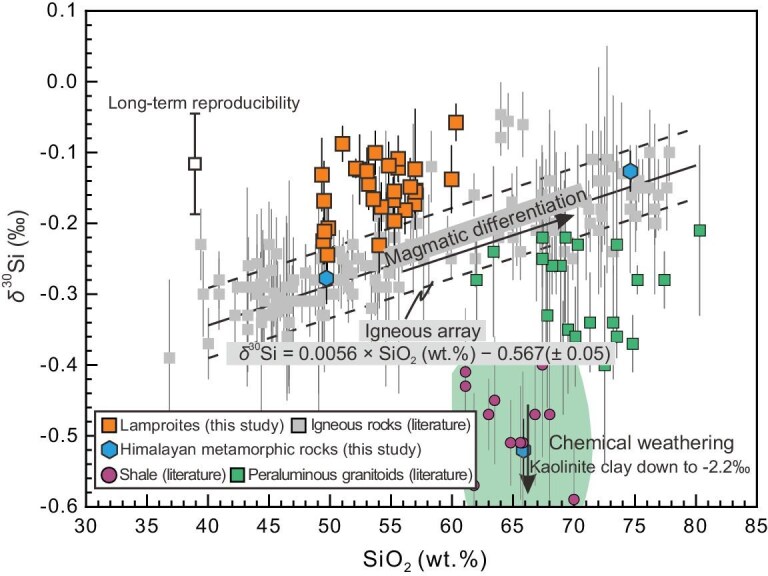
δ^30^Si vs SiO_2_ for the lamproites and Himalayan metamorphic rocks analyzed in this study. Uncertainties are expressed as two standard errors (2SE). The igneous array (solid line) and its 2SE envelope (dashed lines), as defined by Savage *et al.* [18], are shown for comparison. Notably, the δ^30^Si values of the lamproites are consistently higher than the predicted values of the terrestrial igneous array at a given SiO_2_ content. For comparison, published data for terrestrial igneous bulk rock samples are also presented. The field of granite data are from Savage *et al.* [20] and Gajos *et al.* [43]; shale data are from Savage *et al.* [40]; clay mineral δ^30^Si data are from Opfergelt *et al.* [16]; other data sources are the same as in Fig. 1.

The Earth’s continental crust is thought to originate from mantle melting, but it is too felsic relative to a mantle-derived mafic melt. Transformation of mafic to felsic composition requires another process involving magnesium (Mg) loss relative to silicon. Magmatic differentiation followed by delamination of mafic–ultramafic cumulates into the mantle, and chemical weathering followed by subduction of soluble elements into the mantle are two important processes in producing Si-rich and Mg-poor continental crust [7,8]. Fractional crystallization of mafic magmas or partial remelting of mafic crust can lead to a slight enrichment of ^30^Si in the derivatives of Si-rich melts [18], such as some I-type granites [20]. However, continental weathering leaves behind Si-rich residues depleted in ^30^Si [16], such as some sediments and their derived S-type granites [20,21]. The counterbalance of these two processes, with opposite trends in silicon isotope variations, may explain why the bulk continental crust and the bulk silicate Earth (BSE) have almost identical silicon isotope compositions (Figs 1 and 2; [22]). Furthermore, the high-temperature mantle melting process responsible for juvenile crust formation does not induce significant silicon isotope fractionation [23,24]. This implies that the mantle should not show any significant silicon isotope anomalies relative to the BSE. However, some ocean island basalts (OIBs) and continental arc magmas have lighter silicon isotopic compositions compared to the BSE (Fig. 1; [25,26]), indicating that there should be a complementary heavy silicon isotope reservoir in the mantle to maintain balance. Previous studies suggest that partial melting of variably silicified basalts or assimilation of authigenic silica-rich marine lithologies (e.g. chert) in the source can lead to the formation of felsic rocks with heavy silicon isotopes [23,27,28]. However, silicon isotopic compositions currently reported in mantle-derived ultramafic and mafic rocks—including direct mantle samples (e.g. peridotites/pyroxenites), as well as their derivative lavas—are either similar to or isotopically lighter than those of the BSE. This knowledge gap significantly hinders our comprehensive understanding of the deep silicon geochemical cycle.

Most previous research has concentrated on the deep mantle (i.e. asthenospheric mantle [23–26,29,30]), leaving the silicon isotopic composition of the uppermost lithospheric mantle largely unexamined. Ultrapotassic mafic–ultramafic rocks, such as lamproites and shoshonites, are derived from extensively metasomatized lithospheric mantle [31–33]. In this study, we present whole-rock and mineral *in situ* silicon isotope data for lamproites and associated mantle pyroxenite xenoliths in the Lhasa terrane of southern Tibet, which, for the first time, reveal a heavy silicon isotope mantle reservoir. This significant discovery offers a unique opportunity not only to investigate the formation mechanisms of this reservoir, but to determine the silicon isotopic composition of the lithospheric mantle and evaluate the potential of silicon isotopes as tracers of recycled crustal materials.

## RESULTS

**Figure 3.**
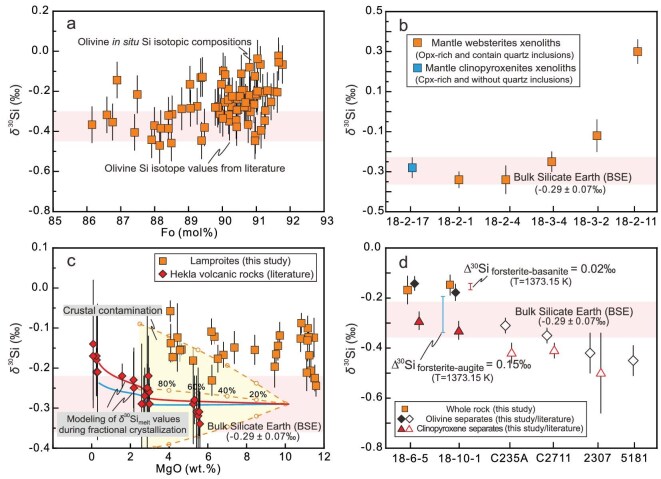
Si isotope compositions of lamproites and associated mantle pyroxenite xenoliths. (a) *In situ* Si isotope analysis of olivine phenocrysts from lamproites exhibits the most distinctive high δ^30^Si signatures yet analyzed. Data sources of olivine Si isotope values are from Georg *et al.* [2], Savage *et al.* [18] and Armytage *et al.* [29]. (b) Opx-rich mantle websterite xenoliths from the lamproites have higher δ^30^Si values than the BSE, whereas Cpx-rich xenoliths show values similar to those of the BSE. (c) Modelling results illustrating the effects of crustal assimilation and fractional crystallization on the lamproites. The wallrock composition (upper continental crust) is defined by SiO_2_ = 66.6 wt.%, MgO = 2.48 wt.% with δ^30^Si = −0.25 ± 0.16‰, representing the end-member compositions for crustal assimilation (from Savage *et al.* [39] and Rudnick and Gao [71]). The yellow shaded region delineates the range of magma δ^30^Si values when the uncertainties (±0.16‰) of the wallrock composition are considered. For comparison, data from Hekla volcano in Iceland [18] are included, where significant Si isotope fractionation was observed during magma differentiation. To assess the impact of different silicate melt compositions, we model the δ^30^Si/^28^Si_melt_ trajectories for basalt (red curve) and basanite (blue curve), utilizing the respective silicon β-factors for each melt type [44]. (d) δ^30^Si values of hand-picked mineral separates from the lamproites. For comparison, silicon isotope data from mineral separates of the Skaergaard Intrusion [18] and Cameroon line spinel lherzolites [2] are also shown.

After the initial India-Asia collision in the Paleocene, numerous post-collisional lamproites were emplaced during the Oligocene–Miocene on the southernmost margin of the Asian continent, the Lhasa terrane in southern Tibet (see Supplementary data for regional geology). The Si isotopic data obtained in this study, along with geochemical data from earlier studies, are reported in Table S1. All samples satisfy the definition of ultrapotassic rocks (MgO > 3 wt.%, K_2_O > 3 wt.%, K_2_O/Na_2_O > 2 [31]) and are lamproites (Fig. S1). Their primitive mantle-normalized incompatible element patterns are distinguished by significantly negative Nb–Ta–Ti and Sr–Ba–P anomalies, resembling the patterns of upper continental crust (Fig. S1). The studied samples have extremely radiogenic initial ^87^Sr/^86^Sr _(t=20Ma)_ ratios (from 0.7160 to 0.7294), negative initial εNd_(t=20Ma)_ (from −15.2 to −12.1) and high (^208^Pb/^204^Pb) _(t=20Ma)_ ratios (from 39.44 to 40.00) at a given (^206^Pb/^204^Pb) _(t=20Ma)_ (from 18.38 to 19.30) compared to pre-collisional arc-related rocks from the same areas (Fig. S2). The rocks under investigation have variable δ^30^Si values ranging from −0.24‰ to −0.06‰ (on average −0.15‰ ± 0.08‰; 2SD; *n* = 33). These values are distinctly higher than those of the BSE, which has a δ^30^Si of −0.29 ± 0.07‰ [22]. Significantly, the samples in this study have consistently heavier δ^30^Si than rocks of the terrestrial ‘igneous array’ at a given SiO_2_ and show the highest δ^30^Si signatures of mantle-derived rocks yet analyzed (Figs 1 and 2). Silicon isotope data obtained from the bulk olivine and clinopyroxene separates from lamproites are as follows: δ^30^Si_olivine_ = −0.16 ± 0.03‰ (*n* = 2), δ^30^Si_clinopyroxene_ = −0.31 ± 0.04‰ (*n* = 2). We also conducted *in situ* silicon isotope analyses of olivine phenocrysts [forsterite (Fo_86–92_)], revealing δ^30^Si values spanning from −0.47 ± 0.09‰ to −0.02‰ ± 0.09 (Fig. 3a).

Some volcanic rocks in this study contain fresh mantle pyroxenite xenoliths (0.5–1.0 cm diameter) with clear boundaries between the xenoliths and their host magmas. Two types of xenoliths (websterites and clinopyroxenites) have been identified. The first type is websterites [orthopyroxene:clinopyroxene (Opx:Cpx) = 55:45], which contain rare, small quartz inclusions (typically <50 μm; Fig. S3a–S3d). The limited occurrence and microscopic scale of quartz and the high Mg^#^ (up to 91) and low TiO_2_ and CaO of orthopyroxene in the websterites suggest a mantle origin, because crustal xenoliths (e.g. granulites) generally have abundant, coarser quartz domains and low Mg^#^ orthopyroxene (Fig. S4). The lack of orthopyroxene phenocrysts in lamproites also precludes a cumulate origin. Rare olivine grains are enclosed by orthopyroxene and exhibit irregular morphologies, likely recording the transformation of olivine to orthopyroxene through melt–rock interaction. Therefore, the minor quartz in the websterites likely reflects localized Si-rich metasomatism in the mantle. Comparable occurrences of quartz-bearing (orthopyroxenite) pyroxenites, characterized by olivine depletion due to metasomatism by silica-rich melts in the mantle, have been reported globally [34,35]. The second type is clinopyroxenite xenoliths, characterized by coarse-grained, euhedral clinopyroxene and are orthopyroxene- and quartz-free, with clinopyroxene exhibiting high Mg^#^ values of 88–94 (Fig. S3e–S3f). The δ^30^Si values of websterite xenoliths range from −0.25‰ to +0.30‰ (*n* = 5), while the clinopyroxene xenoliths show values similar to those of the BSE (−0.28 ± 0.05‰; *n* = 1) (Fig. 3b).

## DISCUSSION

### Influence of crustal processes on Si isotopes

Several crustal processes could potentially contribute to variable δ^30^Si of the lamproites, including hydrothermal alteration or weathering, fractional crystallization and assimilation of materials from wallrock. Thus, it is necessary to evaluate the influence of these processes before discussing the Si isotope composition in the mantle source.

It has been observed that secondary processes, such as seafloor alteration and terrestrial clay mineral formation, leave behind residual materials that are isotopically light, while heavier dissolved silicon is released into the oceans [36]. This process accounts for the lighter silicon isotopes observed in significantly altered basalts and serpentinized ultramafic rocks from the ocean floor [37,38]. Thus, low-temperature alteration cannot explain the high δ^30^Si values of our samples. Moreover, most of our samples are fresh, with loss on ignition (LOI) of <2 wt.%, and show good preservation of primary minerals (Fig. S5). Their δ^30^Si values are neither correlated with LOI nor the chemical index of alteration (CIA) (Fig. S6), indicating that low-temperature alteration does not significantly affect the observed isotopic compositions.

The average Si isotopic compositions of the upper, middle and the lower continental crust are −0.25 ± 0.16‰, −0.23 ± 0.04‰ and −0.29 ± 0.04‰, respectively [39,40]. These values suggest that the overall isotopic composition of the continental crust (−0.26 ± 0.05‰, with weighting methodology detailed in Fig. 1) is remarkably similar to that of the BSE (−0.29 ± 0.07‰ [22]). Even using the extremely heavy Si isotopic compositions of high-Si granites (up to −0.1‰ [21,41]), simple binary mixing between mid-ocean ridge basalt (MORB)-like components and crustal components would require contamination with at least 40%–60% of bulk granitic crust to account for the high δ^30^Si values (Fig. 3c). However, these mixing trajectories do not pass through the primitive lamproites and such a large percentage of assimilated crustal material is incompatible with their high MgO abundances. Importantly, some volcanic rocks contain abundant mantle xenoliths and olivine mantle xenocrysts, suggesting rapid ascent of the magmas and thus a short residence time at crustal levels. Taken together, from these observations it is clear that crustal assimilation was not a major contributor to the high δ^30^Si values in the lamproites.

The influence of magma differentiation on the Si isotopic compositions of basaltic lavas has been extensively studied [18,21,42,43]. In general, the segregation of mafic phases like olivine and pyroxene will result in enrichment of heavy Si isotopes in the residual melts, while the removal of felsic phases like plagioclase and quartz from melts may drive the δ^30^Si of the remaining magmas towards lower values [18,44]. However, there is no significant correlation between δ^30^Si and SiO_2_ or MgO concentrations across the entire dataset, nor within any single volcano (Fig. 3c, Fig. S7). This is seemingly at odds with the predicted relationship between SiO_2_ content and δ^30^Si for igneous rocks, which have been generally thought to have a linear relationship (i.e. igneous array, δ^30^Si = 0.0056 × SiO_2_ − 0.568 [18]). Previous studies have shown that Si isotope behavior during magmatic evolution depends on the mineral phases involved in fractional crystallization and mineral–melt fractionation factors [18,44]. Consequently, liquid lines of descent (LLDs) defined by contrasting melt compositions and physical conditions of crystallization may have distinctly different δ^30^Si_melt_–SiO_2_ trajectories. Modeling of the δ^30^Si_melt_ vs MgO trend shows that a closed-system fractionation model cannot fully explain the δ^30^Si difference (∼0.15‰) between the lamproite and the BSE (Fig. 3c; see Supplementary data for details).

To further constrain possible Si isotope fractionation during magmatic differentiation, olivine and clinopyroxene separates from lamproites were selected for Si isotopic measurements. Olivine is generally the earliest crystallizing phase in mafic igneous rocks and therefore has the potential to record information about primary melts. Our results show that both olivine and clinopyroxene in the lamproites have elevated δ^30^Si values compared to those reported in the literature (Fig. 3d). The difference in δ^30^Si value between olivine and clinopyroxene in the lamproites is consistent with theoretical equilibrium fractionation factors [44] and is similar to that measured in the Skaergaard ultramafic layered intrusion (Fig. 3d; [18]). This observation provides evidence that olivine from lamproites is in Si isotopic equilibrium with other coexisting silicate minerals like clinopyroxene. Utilizing the theoretical equilibrium fractionation factors between forsterite/augite and basanite at 1200°C, the melts in equilibrium with forsterite and augite are estimated to have δ^30^Si of −0.10‰ and −0.09‰, respectively. This suggests that the parental magmas were enriched in ^30^Si compared to typical mantle-derived melts (−0.29 ± 0.07‰ [22]).

To further constrain the Si isotopic composition of the primitive magma, we carried out *in situ* microanalyses of Si isotopes in olivine phenocrysts. Our results reveal a consistent isotopic signature between bulk olivine and clinopyroxene separates and *in situ* measurements, both indicating significantly heavier Si isotopic compositions compared to typical mantle values. Notably, we found no discernible correlation between the olivine Fo content and its Si isotopic composition and even olivine with high Fo values (up to 91) retains these heavy Si isotopic characteristics (Fig. 3a). Therefore, we conclude that the elevated δ^30^Si values of lamproites reflect the composition of mantle-derived primary magmas, rather than contamination of isotopically heavy crustal components or fractional crystallization of isotopically light minerals.

### The contribution of slab melts to the heavy silicon isotope mantle reservoir

The analogous Si isotope signatures between komatiites and MORBs—generated by varying degrees of mantle partial melting—and mantle peridotites imply negligible Si isotope fractionation during mantle melting processes [23,24]. Therefore, the higher δ^30^Si of lamproites relative to the BSE requires the addition of materials with heavy Si isotopes to the magma source. The highly enriched trace-element and radiogenic isotope compositions of post-collisional lamproites suggests the presence of recycled crustal material in their mantle sources. However, the nature of the crustal material responsible for the high δ^30^Si of lamproites and the form in which it is recycled into the mantle remain unclear.

Modification of the mantle by subducted oceanic sediments has previously been invoked to explain the origin of the lamproites in southern Tibet [45]. Seawater and deep-sea siliceous rocks (e.g. diatoms and chert) are isotopically heavy, exhibiting high δ^30^Si values of up to +2‰ [19]. The incorporation of these fluids and siliceous sediments into mantle peridotites may contribute to the formation of lamproites with elevated δ^30^Si [21,46]. However, diatoms did not become abundant until the late Cenozoic [47], while the Neo-Tethyan Ocean between the India and Lhasa terranes closed during the Late Cretaceous to Early Paleocene (65–60 Ma [48]). This indicates that the oceanic sediments at that time likely contained minimal biogenic silicon with heavy silicon isotopes, but rather predominantly consisted of sponge-derived material with light silicon isotopes, a conclusion supported by the extremely light silicon isotope compositions observed in Paleozoic continental arc magmas [25] (Fig. 1). Furthermore, the Phanerozoic oceanic crust included only a small amount of seawater-derived cherts due to the biological removal of silicon from the oceans [49]. Added to this, chert from trench-fill sedimentary units that formed during subduction of the Neo-Tethyan oceanic slab have insufficiently enriched Sr–Nd–Pb isotope signatures to explain the isotope variations of ultrapotassic rocks (Fig. S2). Similarly, chert and radiolarite sedimentary units sampled in ODP Hole 801 also have depleted Sr–Nd–Pb isotope signatures relative to lamproites [50]. Numerous studies have shown that the mantle beneath the Lhasa Terrane, enriched by the subduction of the oceanic slab between 100 and 45 Ma, have more depleted signatures than the source of the post-collisional lamproites [51–53]. This suggests that the enriched component in the mantle source of the lamproites cannot be attributed to materials derived from the Neo-Tethyan oceanic sediments.

**Figure 4.**
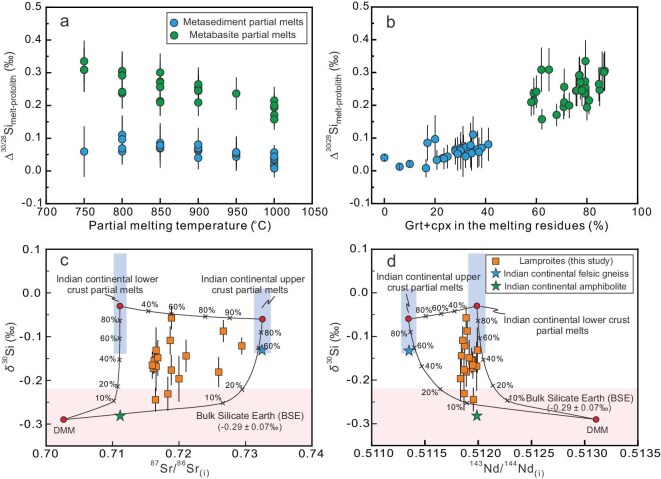
Si isotope fractionation during slab melting and its role in forming a heavy silicon isotope mantle reservoir. (a) Variations of Δ^30/28^Si_melt__–__protolith_ (the difference in δ^30^Si between the partial melt and the protolith) with melting temperature. The data clearly show that, regardless of the melting temperature, the degree of silicon isotope fractionation during metabasite melting is consistently greater than that during metasediment melting. The uncertainty on the mineral–melt Si isotope fractionation factor was taken as the source of uncertainty in the melting models. (b) Relationship between Δ^30/28^Si_melt__–__protolith_ and the proportion of garnet + Cpx in the melting residues. The data suggest that the presence of these minerals (which is enriched in light Si isotopes) in the residual phases significantly influences silicon isotope fractionation, with higher proportions leading to greater fractionation. (c and d) Three end-member mixing models describing the effect of slab melts on Si isotopes of the mantle wedge. The solid curves represent the calculated mixing lines between the depleted MORB mantle (DMM), melts derived from the Indian continental upper crust, and melts derived from the Indian continental lower crust. The percentage values marked on the tick marks of these curves correspond to the proportion of melt originating from the Indian continental crust. The detailed calculations are outlined in the Supplementary data.

High Li contents of the olivine from our samples indicate the recycling of continental crustal material into the mantle source [54] (Fig. S8). Additionally, seismic tomographic images reveal that the Indian continental lithosphere has been underthrusted beneath southern Tibet [55], reinforcing the close relationship between continental subduction and mantle metasomatism. Due to the low mobility (i.e. concentration) of Si in aqueous solutions [56], we favor a melt-mediated transfer of silicon from the subducting continental crust to the mantle wedge. This is also supported by the low Ba/Th ratio and high (La/Sm)_N_ ratio of lamproites (Fig. S9). We used experimentally determined melting reactions of basaltic and granitic lithologies coupled with mineral–melt Si isotope fractionation factors to model the magnitude of Si isotope fractionation that occurred during slab melting (Fig. 4a and b; see Supplementary data for details). The phase proportions of clinopyroxene, garnet, phengite and quartz—key reservoirs of silicon in the melting residues of metabasalt and metasediment—play a critical role in controlling the degree of Si isotope fractionation during slab melting. For example, when basaltic slabs undergo partial melting (clinopyroxene_1_ + garnet_2_ ± kyanite ± quartz/coesite = melt + clinopyroxene_2_ + garnet_2_), the residues are dominated by clinopyroxene and garnet, which are enriched in light Si isotopes, resulting in relatively large Si isotope fractionation (up to +0.33‰; Fig. 4b). In contrast, melting of metasedimentary rocks (phengite_1_ + quartz/coesite_1_ ± clinopyroxene_1_ = melt + phengite_2_ + quartz/coesite_2_ ± garnet ± clinopyroxene_2_), where phengite and quartz (which is enriched in heavy Si isotopes) are major residual phases in addition to clinopyroxene and garnet, leads to a smaller fractionation of Si isotopes (up to +0.11‰; Fig. 4b).

A natural felsic gneiss sample (TB–1) and an amphibolite sample (YLXB–34) were collected from the Higher Himalayan and these were taken to represent the typical upper and middle crustal lithologies of subducted Indian continental slab. Using the modelled magnitude of Si isotope fractionation during slab melting, we suggest that partial melts of a felsic gneiss and an amphibolite is characterized by heavier Si isotope compositions compared to their protoliths, with δ^30^Si values ranging from −0.14‰ to +0.09‰ and from −0.14‰ to +0.01‰, respectively (Fig. 4c and d). Combined with published Sr–Nd isotope results, modeling calculations indicate that the addition of approximately 20%–80% continental crust-derived, ^30^Si-rich melts to the mantle wedge is necessary to explain the Si–Sr–Nd isotopic compositions of the ultrapotassic rocks (Fig. 4c and d). Such a high proportion of continental crust-derived melts can be reasonably achieved because interaction of mantle peridotite with melts derived from subducted continental crust would lead to the formation of mantle domains containing phlogopite- and pyroxene-rich veins within peridotite wall rocks [57]. Due to the lower solidus temperature of pyroxene-rich veins compared to that of peridotite wall rocks, they contribute significantly to the ultrapotassic melt budget. Furthermore, reaction experiments have shown that many of the key features of lamproites (e.g. high K_2_O, Ba, and K_2_O/Al_2_O_3_) would be lost if vein-derived lamproitic melt further interacted extensively with wallrock peridotite [58].

A significant contribution of melts from a phlogopite-bearing pyroxenite source for the lamproites in southern Tibet is also demonstrated by: (i) olivine phenocrysts of the lamproites with low calcium (Ca), titanium (Ti) and high lithium (Li) contents, comparable with those of partial melts of pyroxenite (Fig. S8); (ii) the geochemical compositions of the lamproites plot in the field for melts derived from pyroxenite (Fig. S8); and (iii) the olivine-free mantle xenoliths found in the lamproites, which are proposed to be the source of the lamproites [59]. Significantly, orthopyroxenes in the websterite xenolith contain quartz inclusions, and their composition is similar to those observed in naturally metasomatized mantle xenoliths and siliciclastic sediment–peridotite reaction experiments (Fig. S4). This similarity indicates that the mantle source has undergone significant silicic melt metasomatism. The Si isotopic composition of the mantle websterite xenoliths reported in this study exhibit a distinctive heavy silicon isotope signature (Fig. 3b). In contrast, clinopyroxenite xenoliths preserve the BSE-like silicon isotope compositions. This dichotomy likely indicates that clinopyroxenites formed through carbonate melt metasomatism [60], whereas the websterites formed by felsic silicate melt interactions [35]. The limited silicon budget of carbonate melts renders them ineffective agents for modifying mantle silicon isotope signatures. Most of the studied lamproites are Si-saturated and andesitic, comparable to the melts of silica-excess pyroxenites (Fig. S10). The minor Si-undersaturation in some samples may result from mixing with melts of silica-deficient pyroxenite or peridotite. Consequently, the mantle source of lamproites in southern Tibet is predominantly silica-excess pyroxenite, which most likely formed through reactions between δ^30^Si-rich silicate melts derived from the subducted Indian continental crust and the surrounding peridotite.

In addition to silicon isotopes reported in this study, isotopes of major elements [Ca, Mg and iron (Fe)] have been used to trace the petrogenesis of lamproites in southern Tibet [45,61]. The substantial contrast in isotopic composition between marine carbonates and the terrestrial mantle enables Ca and Mg stable isotopes to identify recycled carbonate materials within the mantle [62]. Lamproites from southern Tibet have δ^26^Mg and δ^44^Ca values lower than those of the mantle, suggesting that the isotopically light Mg signatures may be due to carbonate-related metasomatism in mantle sources [61]. This interpretation aligns with the discovery of clinopyroxenite-rich pyroxenite xenoliths and the high CaO content in olivine phenocrysts observed in this study (Fig. S8). Iron isotopes are typically utilized to trace lithological heterogeneity in the mantle, as pyroxenite melts display heavier δ^57^Fe values than peridotite melts [63]. It has been found that lamproites in southern Tibet have δ^57^Fe heavier than the BSE, indicating contributions from pyroxenite in the mantle [61]. This is also consistent with the significant contribution of melts from pyroxenite with a heavy Si isotopic composition and high nickel (Ni) content of olivine phenocrysts observed in this study. It follows that the use of these four isotope systems likely provides better constraints on the nature of the mantle source than any one system in isolation.

### Implications for deep Si cycle

An increasing body of evidence from arc geochemistry and experimental petrology indicates that slab melting is a widespread and significant process in modern subduction zones [64,65]. Our modeling results indicate that partial melting of the subducting slab produces melts with a heavier silicon isotope composition compared to the protolith (Fig. 4a and b). If the slab itself is anomalously enriched in heavy silicon isotopes, more extreme heavy Si isotope signatures may be produced in the slab melts, as seen in tonalite–trondhjemite–granodiorite (TTG) suites, where extensive deposition of cherts on the Archean seafloor has contributed to their heavy Si isotope composition [23,27]. Hence, silicic slab melts are recognized as a source of silicon with elevated δ^30^Si values.

The contribution of slab melts to mantle-derived mafic magmas is significantly influenced by the thermal state of the mantle wedge. In continental subduction zones, the mantle wedge typically consists of cold subcontinental lithospheric mantle (SCLM). Slab-derived melts enriched in heavy silicon isotopes, formed by partial melting of the subducted crust, are often completely consumed through reaction with peridotite. This leads to the formation of high-δ^30^Si pyroxenite veins within the lithospheric mantle. When these metasomatic veins are later preferentially melted, they can generate high-δ^30^Si alkaline basaltic lavas, such as the lamproites reported in this study. By contrast, in oceanic subduction zones, the contribution of slab melts to the compatible elements and some major elements (including Si) in mafic arc magmas may be diluted by peridotite melts. This occurs because water-rich slab melts can trigger high-degree melting of peridotite as they pass through a hot asthenospheric mantle wedge [66]. Modern adakites provide an exceptional example of slab melts that experience minimal modification as they pass through the relatively cold mantle wedge [67,68]. Adakites are particularly associated with the subduction of hot slab, e.g. young oceanic crust or old oceanic crust with slow convergence rates, where hotter-than-average slabs and colder-than-average overlying mantle result in a greater proportion of slab melt and a reduced contribution from mantle wedge melting [64,68]. Consequently, adakites retain the anomalously high δ^30^Si values of slab melts [23].

Other unique environments may also harbor reservoirs of heavy Si isotopes in oceanic subduction zones. In the forearc region where the surrounding mantle is relatively cool, the heavy silicon isotope signature of slab melts can be preserved in fertile, metasomatized rocks formed through melt-rock reactions [69] (similar to the continental subduction zones scheme outlined above). These metasomatic rocks may subsequently undergo low-degree melting due to thermal disturbances—such as slab rollback or steepening [70]—resulting in mantle-derived alkalic magmas with heavy Si isotopic signatures. Moreover, the deep recycling of residual slab material after partial melting could contribute to ocean island basalts with low δ^30^Si values [26]. While further investigations are needed, including high-precision Si isotope measurements of global primitive arc lavas with varying mantle wedge thermal structures and sub-arc mantle xenoliths, we propose that reservoirs of heavy silicon isotopes in the mantle are likely widespread. As such, δ^30^Si could serve as a promising tracer for slab melting processes. Our findings also suggest that the slab melt stored in the mantle in the form of metasomatic veins represents a heavy silicon isotope mantle reservoir.

## Supplementary Material

nwaf410_Supplemental_Files
