# Supplementary material for: Discovery of a heavy silicon isotope mantle reservoir
Source: Natl Sci Rev. 2025 Sep 24;12(11):nwaf410. doi: 10.1093/nsr/nwaf410 (PMC12658886; doi:10.1093/nsr/nwaf410)
Supplement: nwaf410_Supplemental_Files [file nwaf410_supplemental_files.zip › Si isotope_SupplementalMaterial_0916v1.docx]

Supplementary Materials for

**Discovery of a heavy silicon isotope mantle reservoir**

Mao-Rui Liu^1,2^†, Jun Wang^1^†, Ze-Xian Cui^1^, Gang-Jian Wei^1,2^, Qing Yang^1^, Yi-Gang Xu^1,2^, Andrew C. Kerr^3^, Derek Wyman^4^, Jiang-Hao Bai^1^, Guan-Hong Zhu^1^, Lin Ma^1^, Lu-Lu Hao^1^, Jin-Sheng Zhou^1^, Jing-Jing Fan^1^, Tong-Yu Huang^1^, Miao-Yan Zhang^1^, Qiang Wang^1,2^*

**Affiliations:**

^1^ State Key Laboratory of Deep Earth Processes and Resources, Guangzhou Institute of Geochemistry, Chinese Academy of Sciences; Guangzhou, 510640, China

^2^ College of Earth and Planetary Sciences, University of Chinese Academy of Sciences; Beijing 100049, China

^3^ School of Earth and Environmental Sciences, Cardiff University; Cardiff, CF10 3AT, UK

^4^ School of Geosciences, The University of Sydney; NSW 2006, Australia

* **Corresponding author.** Email: wqiang@gig.ac.cn (Q. Wang)

† These authors contributed equally to this work.

**The PDF file includes:**

Materials and Methods

Figs. S1 to S13

**Other Supplementary Materials for this manuscript include the following:**

Data files S1 to S6

**Materials and Methods**

**Regional geology and sample information**

The Tibetan Plateau was formed by accretion and amalgamation of several terranes that had drifted northward from the Gondwana supercontinent since the Paleozoic [1]. It comprises the following broadly east-west trending terranes: Qilian, Kunlun–Qaidam, Songpan-Ganze, Qiangtang, Lhasa, and Himalaya terranes from north to south, sequentially separated by the Anyimaqen-Kunlun-Muztagh, Jinsha, Bangong-Nujiang, and Indus-Yarlung Tsampo sutures, respectively (Fig. S11). The Lhasa terrane developed an Andean-type active continental margin formed by the northward subduction of the Neo-Tethyan oceanic lithosphere prior to the collision with Indian continent in the Cenozoic marked by the Indus-Yarlung-Zangbo suture zone (Fig. S11) [2]. Arc magmatic activity in the southern margin of the Lhasa terrane lasted from middle-late Triassic to early Paleocene [2]. The magmatism commenced again in Oligocene-Miocene, and mainly consisted of potassic-ultrapotassic rocks and high Sr/Y felsic rocks [3,4]. There are at least 13 volcanic fields that record mafic alkaline eruptions in the Lhasa terrane, with most of these being located along N–S-trending rifts and with eruption ages of 25–8 Ma [5].

In this study, we measured Si isotopic compositions for twenty-nine ultrapotassic volcanic rocks with good spatial coverage of ultrapotassic magmatism across the Lhasa terrane (Fig. S11). One gneiss and one amphibolite from High Himalays are also included to aid in examining the contribution from subducted Indian continental crust. In order to eliminate the effects of fractional crystallization and contamination, all samples considered here have Mg^#^ > 50 (molar 100×Mg/(Mg+Fe)) and 4–12 wt.% MgO. As emphasized by Wang et al. [6], the use of a higher MgO screen, which is commonly applied to mantle-derived melts, would implicitly assume a peridotite source, which is inappropriate for a source dominated by low-Mg and olivine-poor mantle rocks (e.g., pyroxenite). Most samples from this study have already been characterized for major and trace elements as well as radiogenic isotopic ratios, allowing a direct comparison with silicon isotopes (Table S1).

**MC-ICP-MS Si isotope analysis of mineral separates and whole rock samples**

Sample dissolution, column chemistry and silicon isotopic analysis were performed at the State Key Laboratory of Deep Earth Processes and Resources (DEEPER), Guangzhou Institute of Geochemistry (GIG), Chinese Academy of Sciences (CAS) in Guangzhou, China. Samples were processed using a HF-free alkali fusion technique following established procedure of Georg et al. [7] and Yu et al. [8]. All procedures were carried out in a clean laboratory. The sample powder (5–10 mg) was well mixed with alkali flux (~ 200 mg of high purity NaOH powder) and heated in a silver crucible (with lid) at 720 °C for 10 mins to produce a soluble metastable silicate. When the crucible cooled, the outside and the bottom of the crucible were carefully cleaned with water to get rid of any possible contamination. The crucible was then placed into a 60 mL Teflon vial with ~20 mL ultrapure water and sit at room temperature overnight. After the crucible was removed from the Teflon vial, enough HNO_3_ was added to the sample solution in the Teflon vial to attain a solution acidity of 1% HNO_3_ (v/v) for column chemistry. Silicon was separated from matrix elements using 10mL Bio-Rad polypropylene columns with 2 mL of Bio-Rad AG50 W - X12 cation exchange resin (200-400 mesh). The resin was cleaned with 6 mol/L HNO_3_, 6 mol/L HCl, 3 mol/L HNO_3_ and ultrapure water prior to sample loading. Then, the sample solution (1 mL) containing ~45 μg Si was loaded into the column. It is important to ensure that the pH of the resin is neutral before the introduction of the sample solution. Silicon was collected immediately after the sample was loaded, and 6 mL of ultrapure water was loaded to further elute Si. All samples and standards were processed through this chemistry before analysis. The Si recovery of each sample was > 98 %, and the total procedural blank was < 60 ng, which was negligible (< 1 ‰) compared with the total loaded Si.

Silicon isotope measurements were performed on a Nu 1700 Multi-Collector Inductively-Coupled-Plasma Mass-Spectrometer (MC-ICP-MS) at the State Key Laboratory of DEEPER, GIGCAS. The interference of ^14^N^16^O^+^ on ^30^Si were fully resolved using the medium resolution setting of the instrument (Fig. S12). The resulting sensitivity was ~15 V for ^28^Si, with a 3 ppm Si solution with wet plasma conditions. Each single analysis consists of one block of 40 cycles (4.194 second integration). Silicon isotopic data are measured using standard-sample bracketing method and are reported as relative deviations from the silica sand standard NBS28 in δ notation according to the following formula:

δ^30^Si_sample_ = [(^30^Si/^28^Si) _sample_ / (^30^Si/^28^Si) _NBS-28_ -1] × 1000

We discuss the Si data in terms of δ^30^Si values which are approximately twice that of δ^29^Si (no mass-independent Si isotope variations have so far been measured in terrestrial systems; [9]). Analytical reproducibility on δ^30^Si is better than ± 0.07‰ (2SD) based on repeated analyses of several international standards against NBS-28 (n>50, Fig. S13) in one year. The measured geological standards BCR-2, BHVO-2 and JA-2, which were purified and analyzed together with samples in this study, yield δ^30^Si values of –0.21 ± 0.07‰ (n=10, 2SD), –0.29 ± 0.07‰ (n=34, 2SD) and –0.13 ± 0.05‰ (n=9, 2SD), consistent with the recommended values [10–12] (Fig. S13). The δ^30^Si differences of sample replicates (separate digestion and measurement) are less than 0.06 ‰ (Table S1–2), within the long-term external reproducibility (~0.07‰).

To ensure the robustness of the data, sample duplicates were produced and additional analyses were performed on a Thermo Scientific Neptune Plus MC-ICP-MS at the CAS Key Laboratory of Crust-Mantle and Environments at the University of Science and Technology of China (USTC). Details about the procedures of sample dissolution, column chemistry and mass spectrometry analyses including instrumental parameters is fully described in Yu et al. [8]. Individual analyses of the ultrapotassic rocks in two laboratories show consistent results (Table S1), confirming the precision and validity of our Si isotopic data.

**SIMS in situ Si isotope analysis of olivine grains**

Olivine grains separated from the lamproites along with standard reference materials (San Carlos and 06JY06 olivine) [13,14] were placed on a double adhesive tape and mounted in Sn-based alloy according to the method of ref [15,16]. In situ silicon isotopic measurements were conducted using a CAMECA IMS 1280-HR secondary ion mass spectrometer (SIMS) at the State Key Laboratory of DEEPER, GIGCAS. A Cs^+^ primary beam of ~7 nA with an impact energy of 10 keV was used to sputter secondary ions from a ~35 μm sample area (15 μm spot size + 20 μm rastering), with a normal-incidence electron gun providing charge compensation. A nuclear magnetic resonance controller was used to stabilize the magnetic field. The entrance slit and the field aperture were set to 200 μm and 5000 μm, respectively, and the energy slit was set to a 40 eV bandwidth and shifted 5 eV below the maximum transmission. Negatively charged secondary ions were accelerated by a voltage of 10 kV. The ^28^Si^-^ and ^30^Si^-^ ions were detected using two Faraday cup (FC) detectors posited at L1 and H’2 with resistors of 10^10^ Ω and 10^12^ Ω, respectively. The FC detectors were equipped with an exit slit of ~500 μm to obtain a broad, flat-topped mass peak, corresponding to a mass resolution power of ~2400 (10% peak height). However, the ^29^Si^1^H^-^ was challenging to separate from the ^30^Si^-^ signal effectively under such a setting, even though the external H contamination was blocked efficiently by making alloy mounts and then long-time pumping in the analysis chamber (2.5 × 10^-9^ mbar). Drawing from the approach of Yu et al. [17], the magnetic field was locked at the left quarter of the flat top of the ^30^Si^-^ signal that could drop the ^29^Si^1^H^-^ intensity by more than two orders of magnitude. Thus, the influence of ^29^Si^1^H^-^ tailing on ^30^Si^-^ can be ignored. Each analysis lasts for ~4.5 min, including 30 s pre-sputtering, automated beam alignment (centering DTFA and DTCA), and integrating 30 cycles of static analysis of ^30^Si/^28^Si. Si isotope data of olivine were normalized to the measured primary standard San Carlos olivine (SCO), which was interspersed in the analytical sequence and has a recommended δ^30^Si value of –0.30‰. Secondary standard 06JY06 olivine separated from mantle peridotite xenoliths yield an average δ^30^Si value of –0.35 ± 0.11‰ (2SD, n = 8), consistent with mantle nature. In addition, we further analyzed three zircon standard samples (Penglai, Qinghu, and SA01) with known Si isotope compositions under the same instrument parameters to assess the accuracy of the analytical procedure. The results showed that the Qinghu and SA01 zircon calibrated with Penglai zircon had Si isotope values of –0.39 ± 0.12‰ (2SD, n = 38, 2 outliers) and –0.19 ± 0.11‰ (2SD, n = 49, 1 outliers), respectively, which is consistent with their recommended values –0.45 ± 0.06‰ (2SD) [17] and –0.24 ± 0.10‰ (2SD) [18]. Previous studies [19] have undertaken detailed work on SIMS matrix effects showing δ^30^Si variations in olivine as a function of Fe molar fractions and demonstrated a complex correlation between instrumental fractionation of ^30^Si/^28^Si and olivine Mg^#^. However, the instrumental mass fractionation of Si isotopic analysis can be neglected [19] within the Mg^#^ range of 70-100. This olivine Mg^#^ interval covers the compositional range of the olivines studied here (Mg^#^: 86-92; Table S3). Therefore, instrumental mass fractionations (IMFs) are negligible for Si-isotope ratios of olivine samples analysed in this work.

**Mineral composition analyses**

Pyroxene and olivine composition were analyzed with a JEOL JXA-iSP-100 Electron Probe Microanalyzer equipped with five wavelength-dispersive spectrometers (WDS) at the Laboratory of Guangzhou Tuoyan Analytical Technology Co., Ltd. Operating conditions for quantitative WDS analyses involved an accelerating voltage of 15 kV, a beam current of 10 nA and a 2–5 µm spot size. The peak counting time was 10 s for K, Na, Fe, Si, Mg, 20 s for Ca, Al, Mn, Ti, Ni and 30 s for Cr. The background counting time was 1/2 of the peak counting time on the high- and low-energy background positions. The following standards were used: Quartz (Si), Olivine (Mg), Orthoclase (K), Rutile (Ti), Magnetite (Fe), MnO_2_ (Mn), Albite (Na), Spodumene (Al), Apatite (Ca), Cr_2_O_3_ (Cr), NiO (Ni).

**Modeling Si isotope fractionation during fractional crystallization**

To model the Si isotopic composition of lamproites during crystallization, we firstly modeled the liquid line of descent of the lamproites using the MCS thermodynamic program [20]. The least differentiated sample from Sailipu volcanic field was chosen as a parental magma (MgO=10.29 wt.% [21]. Based on published calculations of the thermobarometer for the Sailipu volcanic rocks, the oxygen fugacity, pressure, and initial water content were set at FMQ +1, 4 kbar, and 1.5 wt.%, respectively [21–23]. Using the calculated phase proportions, Si concentration of the melt and residual phases, crystallization temperature and Si isotope fractionation factors (Table S4–5), we modeled Si isotope variations in the melt:

$\delta^{30/28}Si_{melt}=(1,000+\delta_{bulk}^{30/28}Si)f^{(\alpha^{30/28}Si-1)}-1,000$ (1)

where $\delta_{bulk}^{30/28}$and $\delta^{30/28}Si_{melt}$ are the starting (bulk) and melt isotopic compositions, $f$ is the mass fraction of silicon in the residual melt, and $\alpha^{30/28}Si$ is the bulk fractionation factor of the fractionating mineral assemblage. The mineral–melt fractionation factors are derived from reduced partition function ratios of minerals and silicate melts based on various theoretical estimates [24–28]. Consequently, it is crucial to evaluate the potential systematic biases among these different calculated β-factors. Notably, the diopside Si β-factor reported by Qin et al. [24] and Li et al. [27] is 8% heavier than that calculated by Rabin et al. [28]. In order to limit the bias due to difference in the calculation methods, we applied a correction of –8% to the mineral Si β-factor (e.g., quartz, albite, anorthite, kyanite, muscovite) from Qin et al. [24] and Li et al. [27] prior to calculating ∆_mineral–melt_ ^30^Si/^28^Si. In Rabin et al. [28], the silicon β-factors for minerals (e.g., forsterite, augite) were computed using Quantum Espresso (QE) codes, whereas the Vienna Ab-initio Simulation Package (VASP) was utilized in Rabin et al. [25] to derive the β-factors for a range of silicate melts (e.g., basanite, basalt and trachyte). As demonstrated by Rabin et al. [25], both computational packages yield comparable results, with discrepancies in the silicon β-factors on the order of approximately 1.3%. We have accounted for this source of error in our calculations. To examine the effect of selecting different silicate melt compositions, we model the trajectories of δ^30^Si/^28^Si_melt_ for basalt and basanite using the respective silicon β-factors for each melt type [25].

**Sr–Nd–Si isotopic three-component mixing model calculation**

The Si isotope composition of Indian continental crust-derived melt is based on measurements of gneiss and amphibolite combined with partial melting modeling conducted in this study. To model the magnitude of Si isotope fractionation during slab melting, we used experimentally determined melting reactions of basaltic and granitic lithologies, coupled with mineral–melt Si isotope fractionation factors derived from first-principle calculations. The starting materials include MORB basalt, radiolarian clay, carbonate-bearing siliciclastic marine sediment, pelite, granitic gneiss, and carbonate-poor sediments [29–36]. The difference in Si isotope composition between the partial melt and the protolith (Δ^30/28^Si_melt–protolith_) can then be calculated through isotopic mass balance (Table S6):

$\delta_{\mathrm{melt}}=\delta_{\mathrm{bulk}}-\frac{\sum\Delta_{i- melt}\times C_{i}f_{i}}{C_{\mathrm{source}}}$ (2)

where δ_bulk_ and δ_melt_ are the starting (bulk) and melt isotopic compositions, Δ_i−melt_ is the equilibrium isotopic fractionation between mineral i and silicate melt, f_i_ is the proportion of mineral i in the total assemblage, and C_source_ and C_i_ are the Si concentrations in the protolith and mineral i, respectively. The sources and corrections of silicon β-factors employed in calculating mineral–melt fractionation factors are consistent with those used in fractional crystallization modeling. The equilibrium Si isotope fractionation between quartz and pyrope was used to calculate ∆_pyrope–melt_ ^30^Si/^28^Si [26].

Trace element forward modeling was carried out using the approach described in Turner et al. [37]. Metasediment melts calculations assume a melt fraction of 30% (aggregated fractional melting, Cl=(Co/F)*(1-(1-F)ˆ(1/D))) based on mean sediment melting partition coefficients from Turner et al. [37]. The modeling work of Turner and Langmuir [38] has found that this melt fraction is most consistent with the range of arc compositions seen in most of the world’s arcs. For metabasite melts, values were computed using melt/starting material enrichment factors from Turner et al. [37] for both "colder" and "hotter" slab melts. The composition of the metasedimentary formations (KR1) [39] and amphibolite (T0394-6) [40] within the Himalayan region were chosen to represent the upper Indian continental crust and lower Indian continental crust, respectively.

The composition of depleted MORB (Mid-Ocean Ridge Basalt) mantle source is as follows [11,41]: Sr = 7.664 ppm, ^87^Sr/^86^Sr(i) = 0.702626, Nd = 0.581 ppm, ^143^Nd/^143^Nd(i)= 0.513106, SiO_2_ = 44.71 wt.%, and δ^30^Si = –0.29 ‰. The initial ratios of Sr-Nd isotopic composition are age-corrected to 20 Ma (average age of the post-collisional, K-rich magmatism in south Tibet). The composition of the slab-derived melt end-member are as follows: upper continental crust-derived melt: Sr = 283.2 ppm, ^87^Sr/^86^Sr(i) = 0.732549, Nd = 11.6 ppm, ^143^Nd/^143^Nd(i) = 0.511343, SiO_2_ = 75 wt.%, and δ^30^Si = –0.06 ‰; lower continental crust-derived melt: Sr = 759.2 ppm, ^87^Sr/^86^Sr(i) = 0.711108, Nd = 16 ppm, ^143^Nd/^143^Nd(i) = 0.511987, SiO_2_ = 75 wt.%, and δ^30^Si = –0.03 ‰.

**Supplementary Figure**


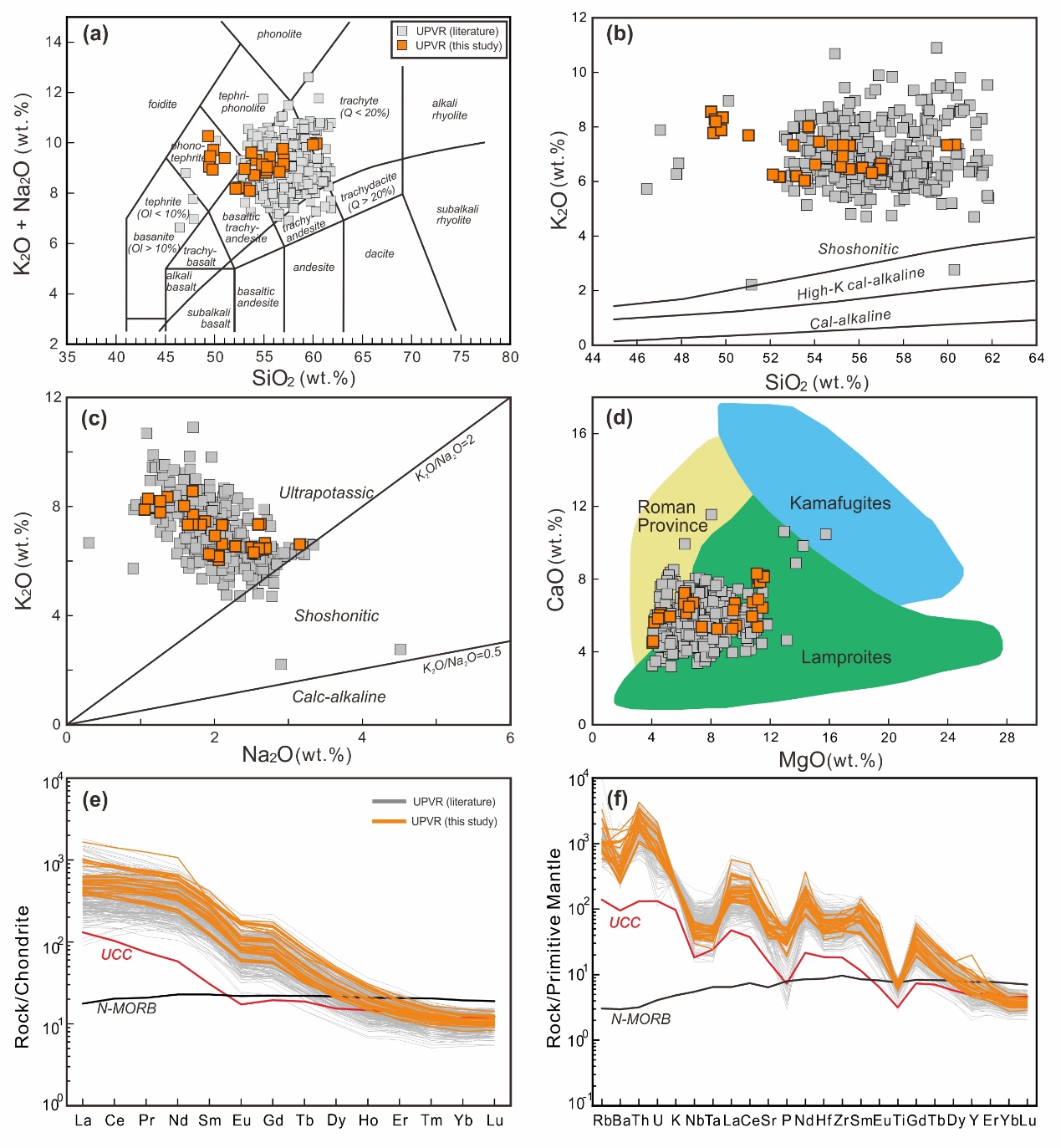


**Fig. S1.** Plots of (a) SiO_2_ (wt. %) versus Na_2_O+K_2_O (wt. %), (b) SiO_2_ (wt. %) versus K_2_O (wt. %), (c) Na_2_O (wt. %) versus K_2_O (wt. %) (d) MgO (wt. %) versus CaO (wt. %), (e) Chondrite-normalised REE and (f) primitive mantle normalized trace-element diagrams for the lamproites. Data source: [4,5,42–57]. Chondrite and primitive-mantle data are from Sun and McDonough [58].


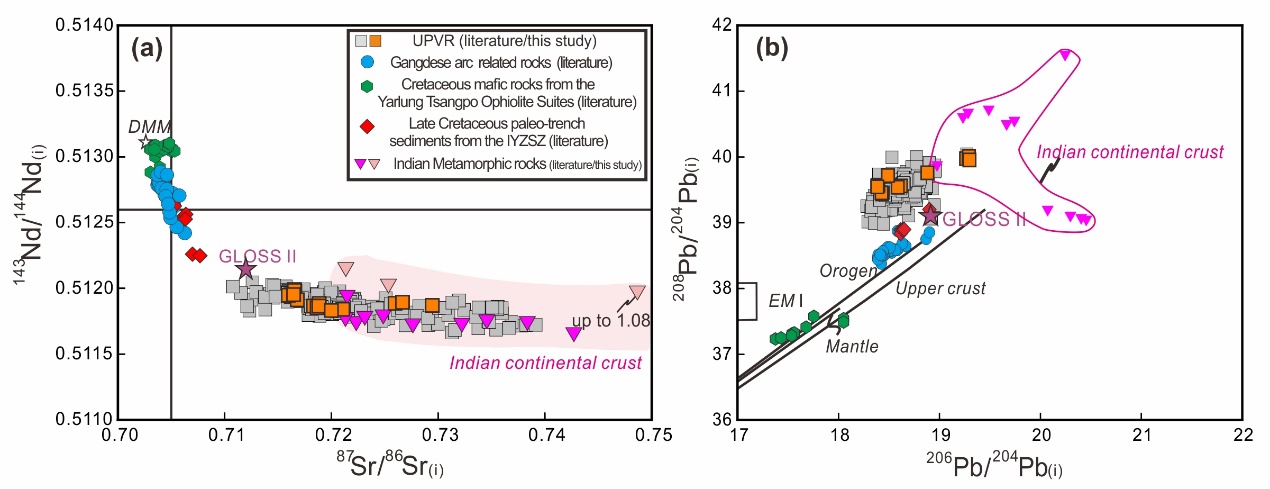


**Fig. S2.** Sr-Nd-Pb isotope plot for post-collisional ultrapotassic rocks in the Lhasa block. Mesozoic Gangdese arc related rocks ([59]; and references therein), Cretaceous mafic rocks from the Yarlung Tsangpo Ophiolite Suites ([60–62]), paleo-trench sediments from the Indus–Yarlung Zangbo suture zone (IYZSZ) [59] and Indian Metamorphic rocks [40,63–65] are also shown for comparison.


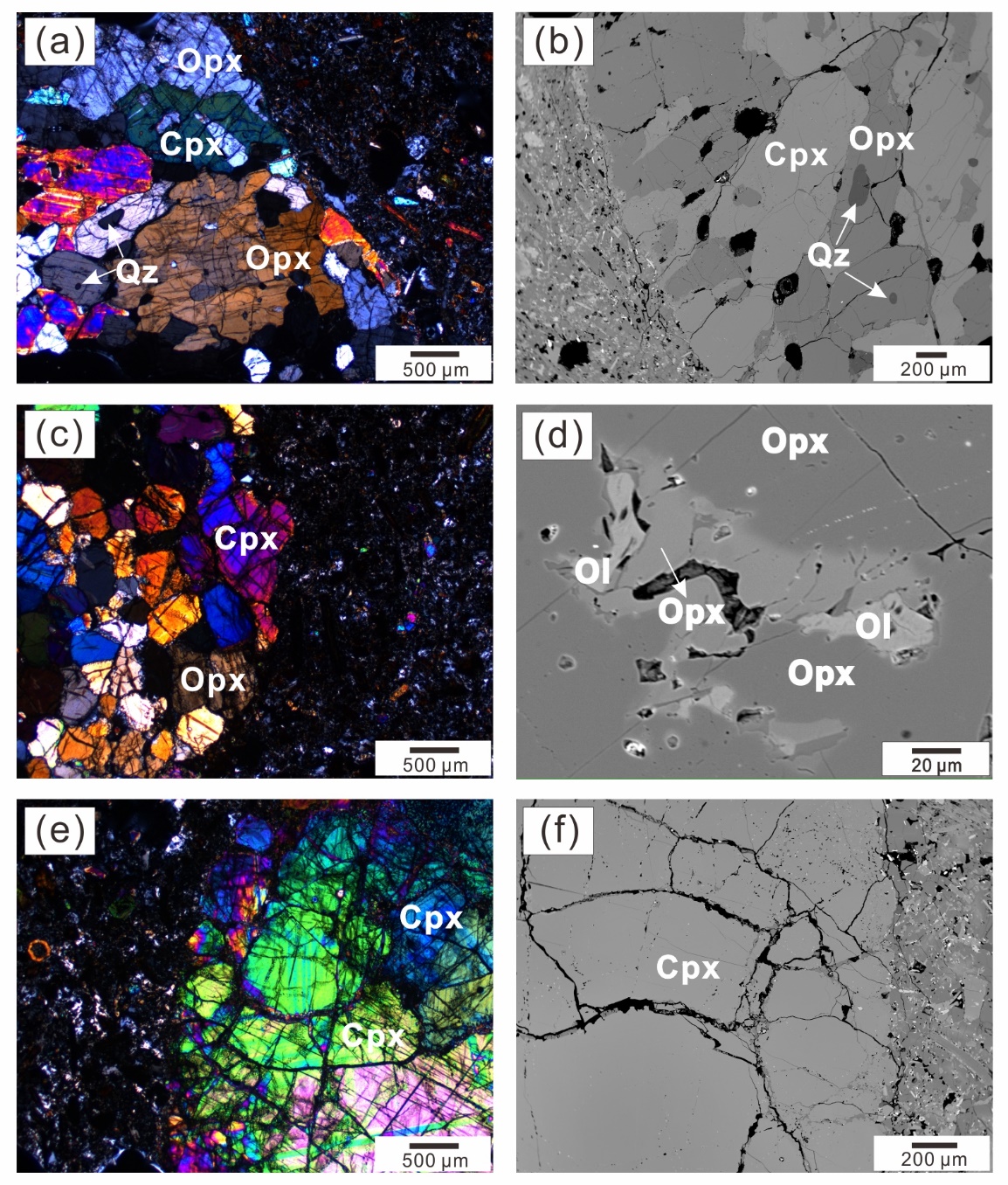


**Fig. S3.** Representative photomicrographs and backscattered electron (BSE) images of the olivine-free mantle xenoliths from Sailipu lamproites. Mantle websterites xenoliths are opx-rich and contain rare, small quartz inclusions (a-d) while mantle clinopyroxenites xenoliths are cpx-rich and without quartz inclusions (e-f). (d) Note that relics olivine is enclosed by orthopyroxene, which likely records the transformation of olivine to orthopyroxene through melt–rock interaction. Abbreviations: Cpx: clinopyroxene; Opx: orthopyroxene; Qz: quartz.


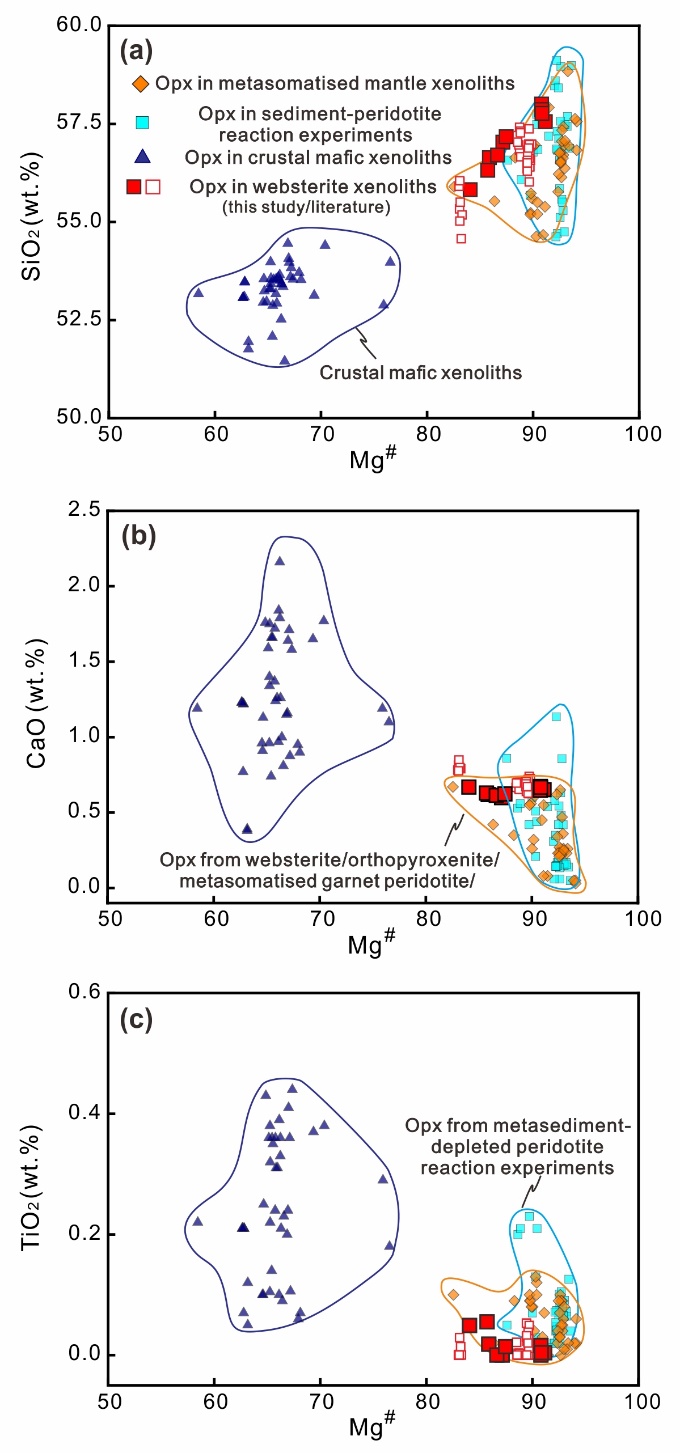


**Fig. S4.** (a-c) Compositional comparison between the high Mg^#^ orthopyroxene from the websterite xenoliths from Sailipu lamproites ([66] and this study) and those from the crustal mafic xenoliths in Tibet [67–69], Opx from natural metasomatised mantle xenoliths [70–77] and sediment-peridotite reaction experiments [31,75,78,79].

**
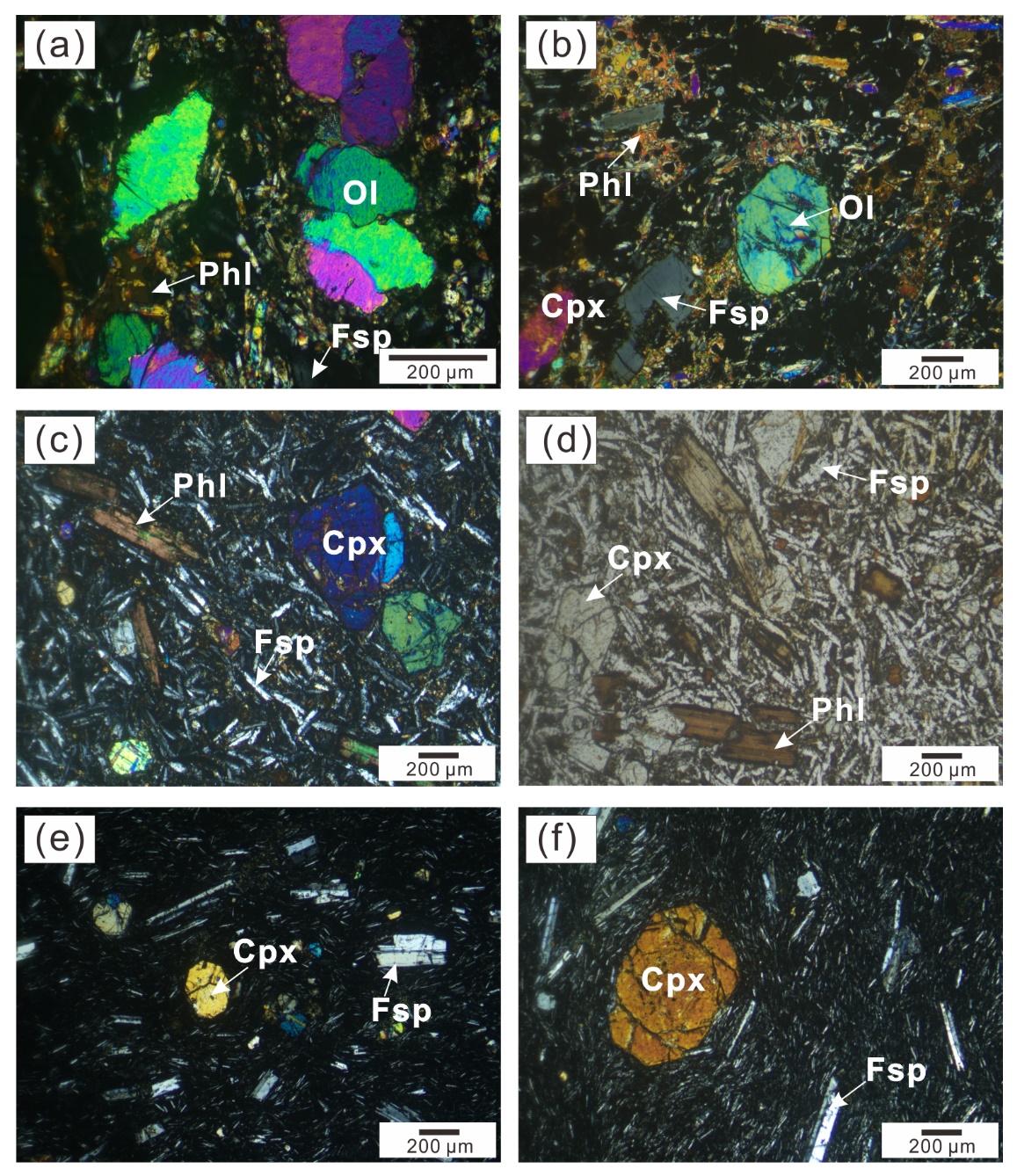
**

**Fig. S5.** Representative photomicrographs of the ultrapotassic rocks. Ol olivine; Cpx clinopyroxene; Phl phlogopite; Fsp feldspar.

**
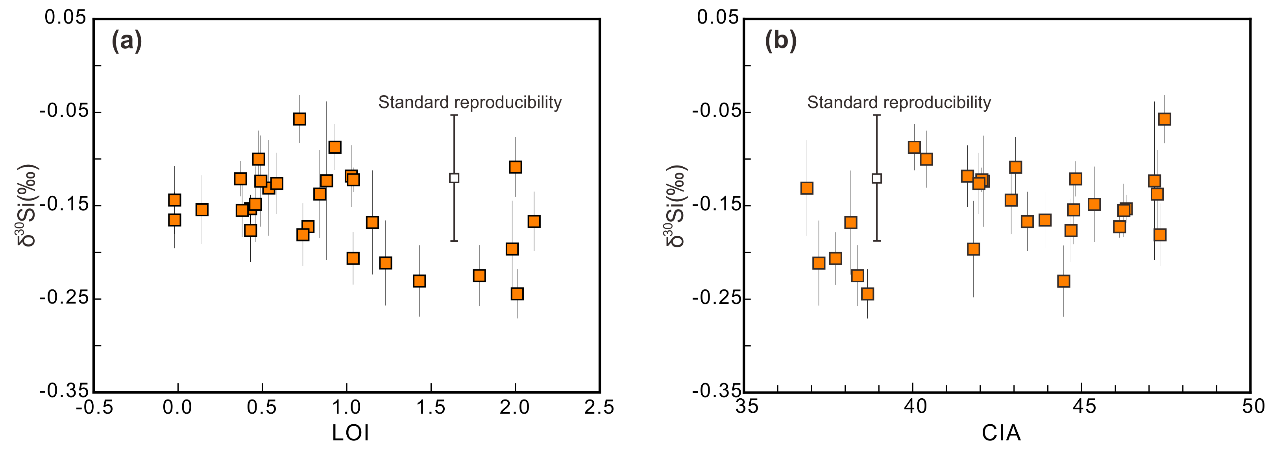
**

**Fig. S6.** δ^30^Si versus (a) LOI (loss on ignition; wt.%) and (b) CIA (chemical index of alteration; defined as Al_2_/O_3_ / (Al_2_O_3_ +CaO + Na_2_O + K_2_O) with oxides given in molar portions).


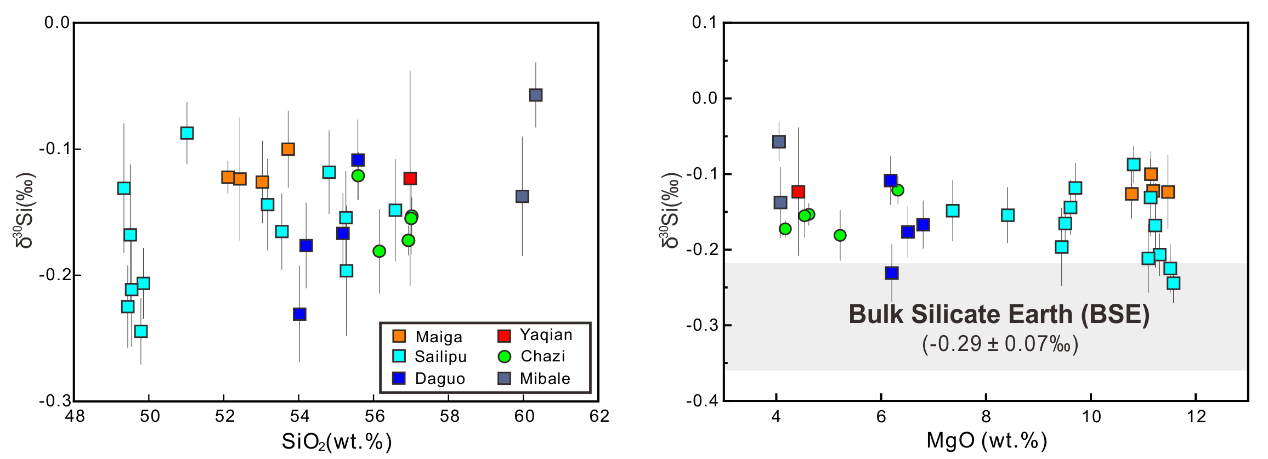


**Fig. S7.** δ^30^Si versus (a) SiO_2_ and (b) MgO of the lamproites analysed in this study, showing that there is no significant correlation between δ^30^Si and SiO_2_ and MgO concentrations across the entire data set, or within any of the groups in the studied volcanic succession.


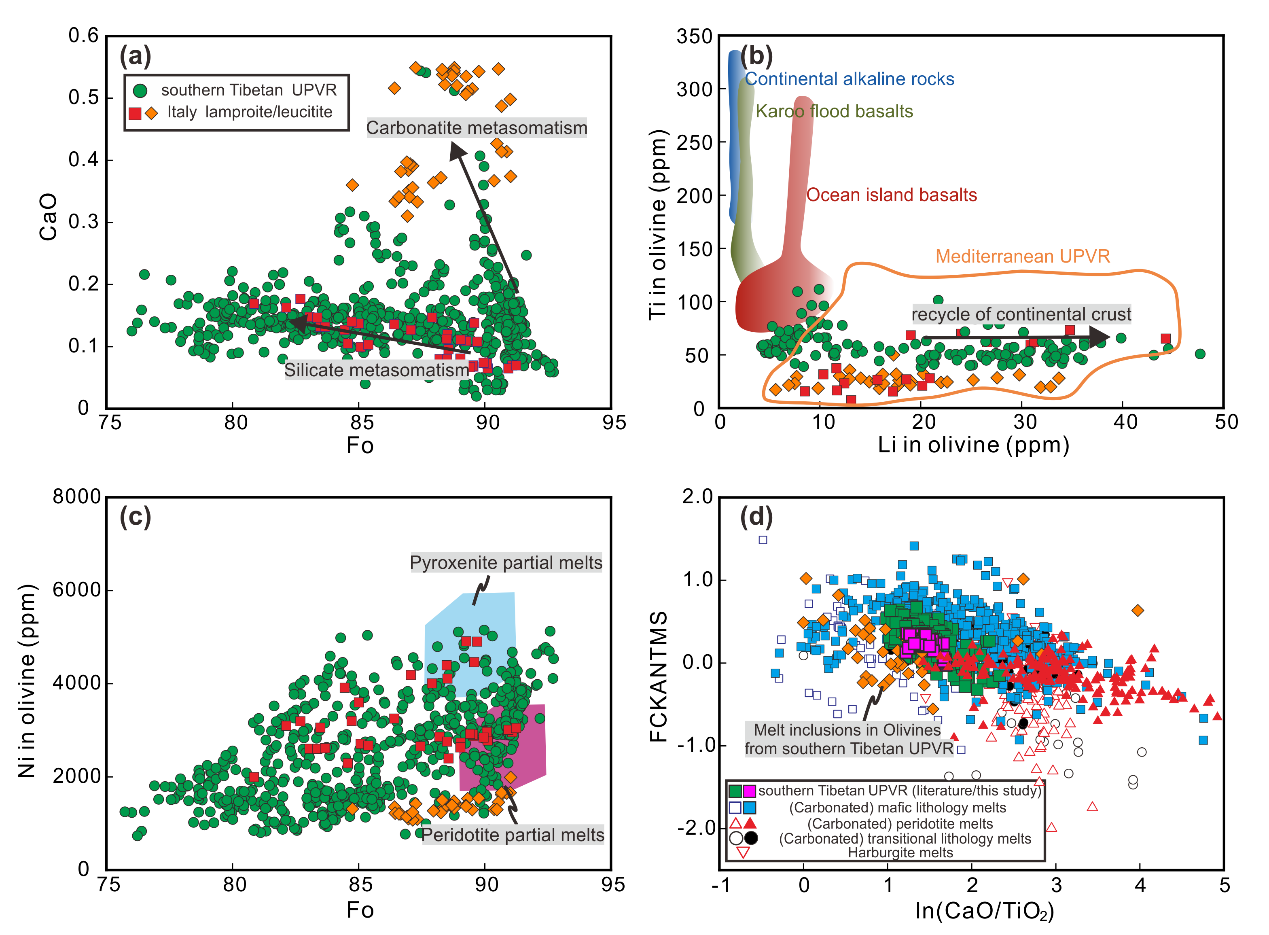


**Fig. S8.** Relationship between olivine Fo (Fo=100*Mg/(Mg+Fe)) and CaO (a), olivine Li and Ti (b), olivine Fo and Ni (c), and bulk rock ln (CaO/TiO_2_) and FCKANTMS (d). Olivine chemistry from Italian lamproites and leucitites is also shown for comparison [80]. A parameter called FCKANTMS is proposed to identify source lithologies for basaltic melts [81]. Experimental data of the partial melts from different mantle lithologies were collected by Yang et al. [81].


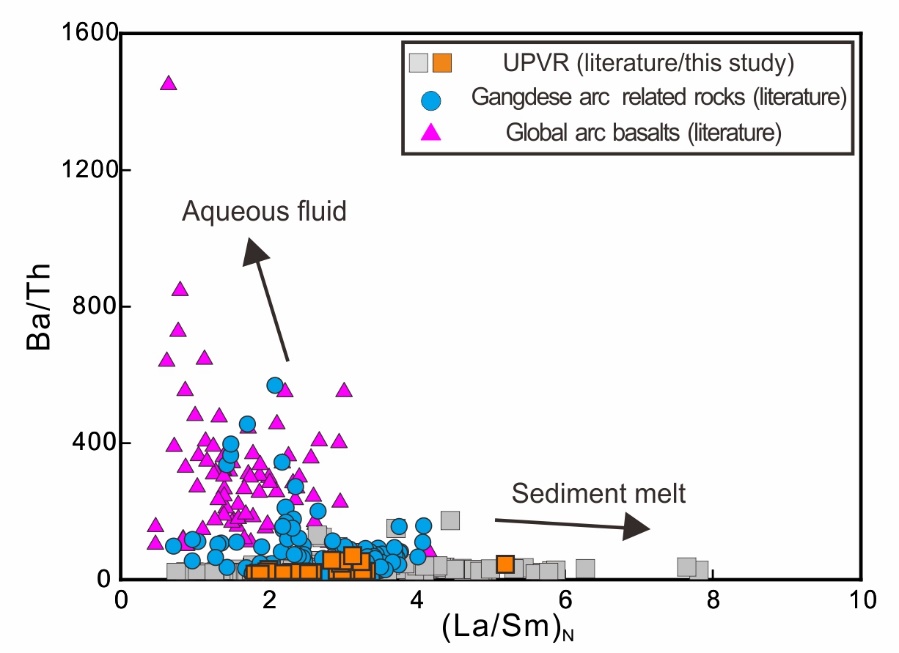


**Fig. S9.** (La/Sm)_N_ versus Ba/Th. Data source: Global arc basalts ([82]; other data sources and symbols are the same as in Fig. S2.


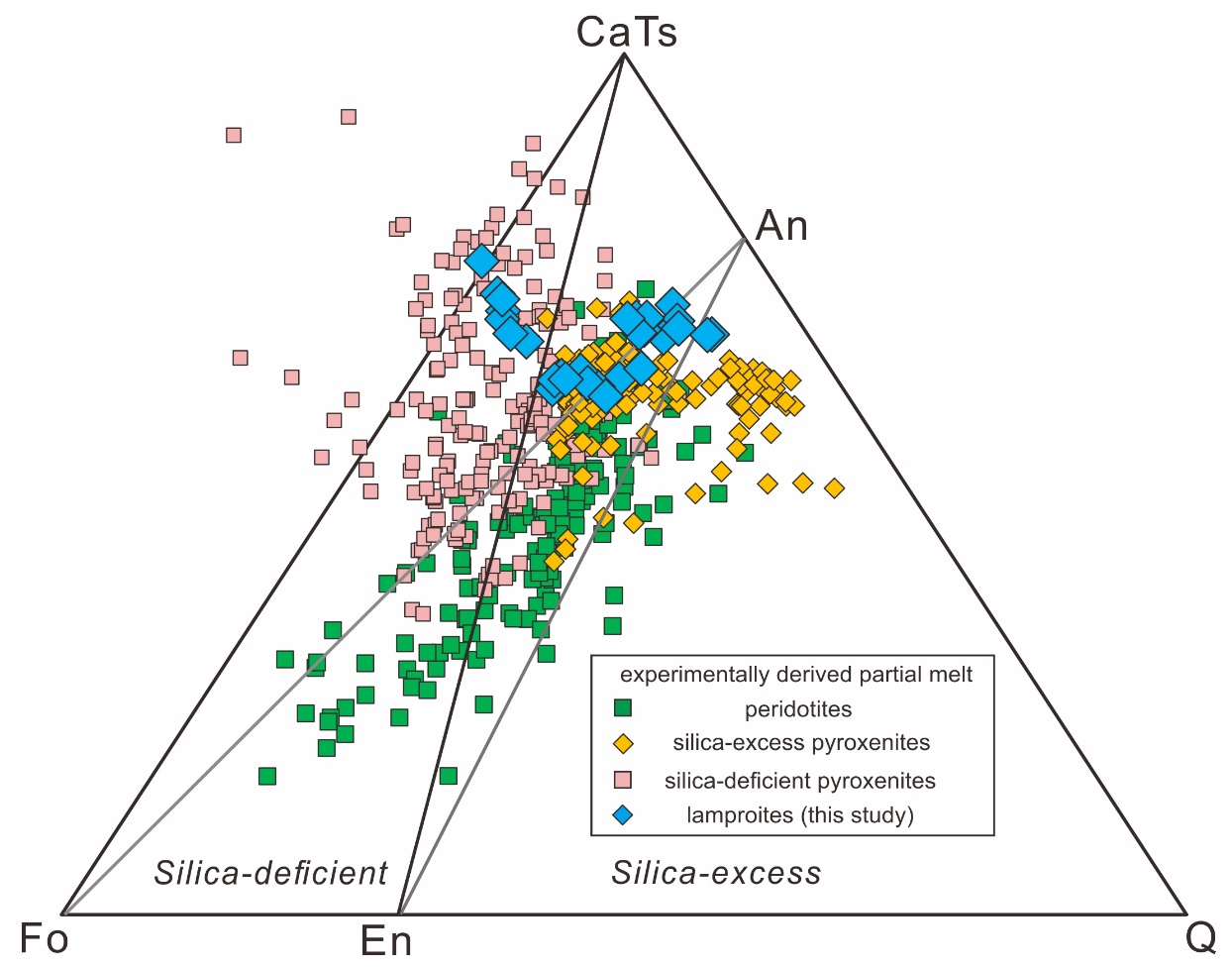


**Fig. S10.** The studied lamproites compared to experimentally derived partial melt compositions of peridotite, silica-excess pyroxenite and silica-deficient pyroxenite on the Ca-Tschermak-Anorthite-Quartz-Enstatite-Forsterite (CaTs-An-Q-En-Fo) plane from Diopside (Di), after the projection scheme of O’Hara [83]. The enstatite (En)–CaTs join separates silica-deficient and silica-excess pyroxenites. Experimental data of the partial melts from different mantle lithologies were collected by Yang et al. [81].


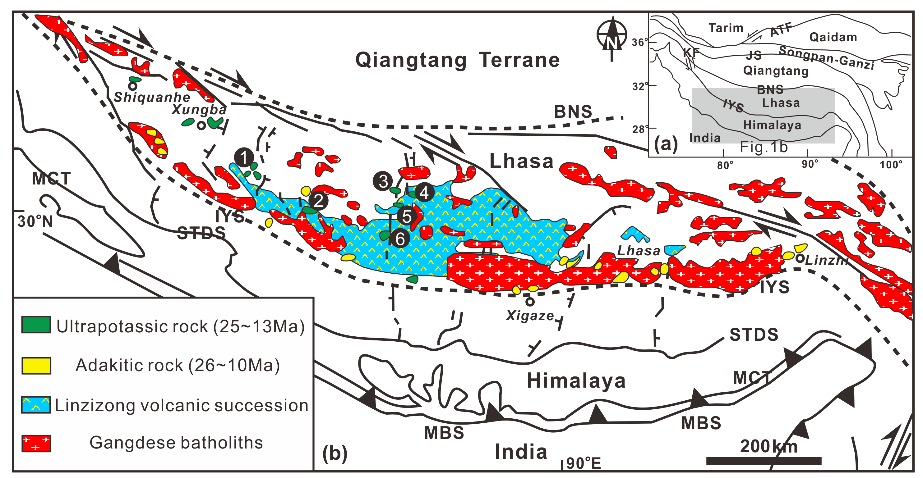


**Fig. S11.** Simplified geological maps of the Tibetan–Himalayan orogen illustrate the distribution of Miocene potassic–ultrapotassic lavas and adakitic granitoids, the Paleocene-Eocene Linzizong volcanic succession, and the Mesozoic Gangdese and related batholiths (modified from Hou et al. [84]). An inset displays the tectonic framework of the orogen. The sampling locations for Si isotopic analysis across six volcanic fields, listed from west to east, are: 1–Sailipu, 2–Maiga, 3–Yaqian, 4–Mibale, 5–Daguo, and 6–Chazi.


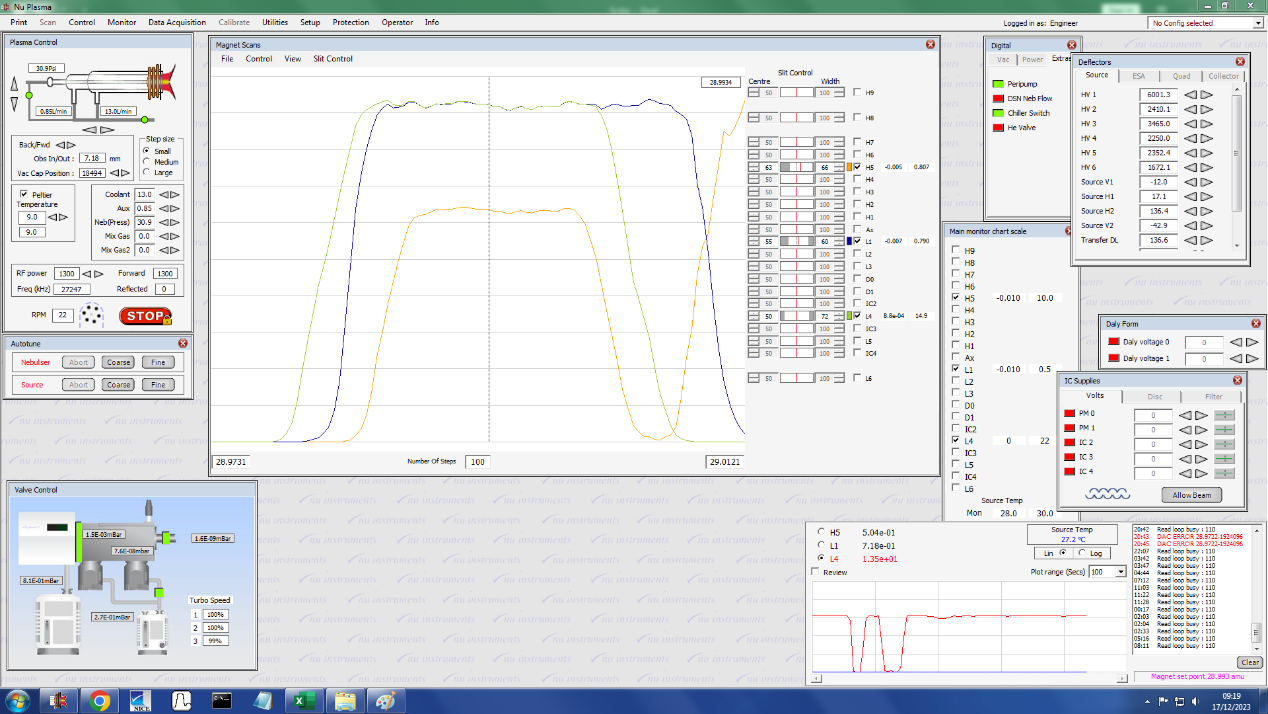


**Fig. S12.** Peak scans of mass 29 on a Nu1700 MC-ICP-MS, showing that the interference of ^14^N^16^O^+^ on ^30^Si were fully resolved.


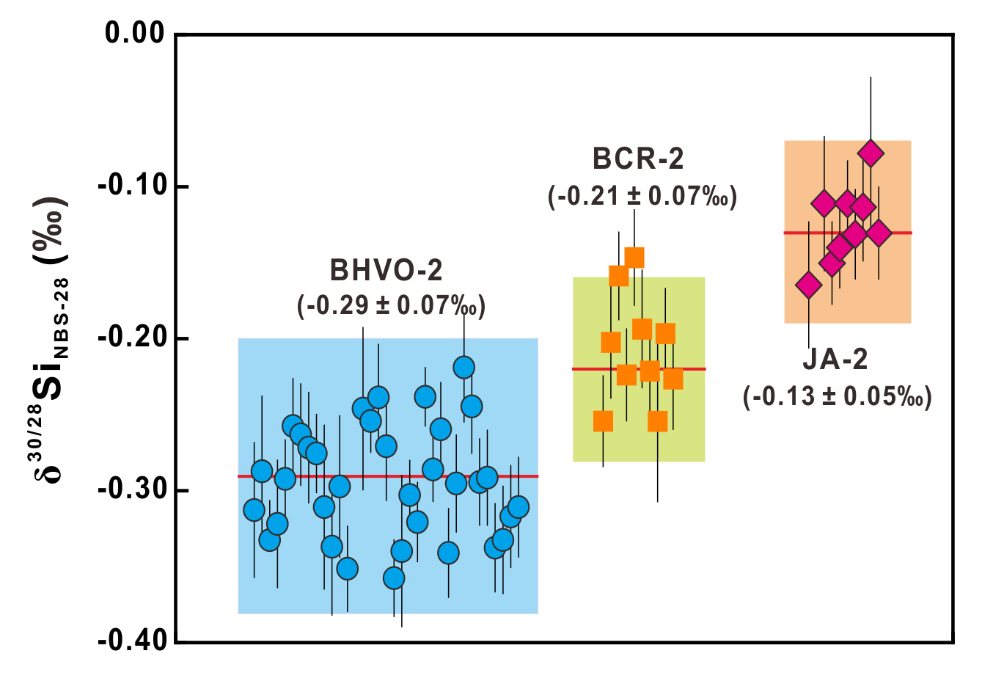


**Fig. S13.** Comparison of δ^30/28^Si values measured in this study (± 2 se) with those reported in literature data. The shaded area encompasses the recommended ranges for BHVO-2, BCR-2, and JA-2, which are –0.29 ± 0.09‰ (2sd, n = 188), –0.22 ± 0.06‰ (2sd, n = 178), and –0.13 ± 0.06‰ (2sd, n = 6), respectively, as reported by Savage et al. [85], Zeng et al. [86], and Zambardi and Poitrasson [87].

**Data S1-S7. (separate file)**

Data S1 - Silicon isotope composition of post-collisional ultrapotassic rocks and associated mantle pyroxenite xenoliths from southern Tibet.

Data S2 - Si isotopic composition of reference materials determined in this study and its corresponding values obtained from published data.

Data S3 - In situ Si isotopic composition of Olivine measured by SIMS

Data S4 - Modeling of δ^30^/^28^Si_melt_ values during fractional crystallization.

Data S5 - Modelling Si isotope fractionation during partial melting.

Data S6 - Major compositions of minerals in mantle pyroxenites xenolith (wt.%)

**REFERENCES**

1. Yin A and Harrison TM. Geologic Evolution of the Himalayan-Tibetan Orogen. *Annu Rev Earth Planet Sci* 2000; **28**: 211–280.

2. Zhu DC, Wang Q, Weinberg RF *et al.* Continental crustal growth processes recorded in the Gangdese batholith, southern Tibet. *Annu Rev Earth Planet Sci* 2023; **51**: 155–188.

3. Chung SL, Liu D, Ji J *et al.* Adakites from continental collision zones: Melting of thickened lower crust beneath southern Tibet. *Geology*. 2003; **31**: 1021.

4. Zhao Z, Mo X, Dilek Y *et al.* Geochemical and Sr–Nd–Pb–O isotopic compositions of the post-collisional ultrapotassic magmatism in SW Tibet: Petrogenesis and implications for India intra-continental subduction beneath southern Tibet. *Lithos*. 2009; **113**: 190–212.

5. Guo Z, Wilson M, Zhang M *et al.* Post-collisional ultrapotassic mafic magmatism in south Tibet: Products of partial melting of pyroxenite in the mantle wedge induced by roll-back and delamination of the subducted Indian continental lithosphere slab. *J Petrol* 2015; **56**: 1365–1406.

6. Wang Y, Foley SF, Buhre S *et al.* Origin of potassic postcollisional volcanic rocks in young, shallow, blueschist-rich lithosphere. *Sci Adv* 2021; **7**: eabc0291.

7. Georg RB, Reynolds BC, Frank M *et al.* New sample preparation techniques for the determination of Si isotopic compositions using MC-ICPMS. *Chem Geol* 2006; **235**: 95–104.

8. Yu HM, Li YH, Gao YJ *et al.* Silicon isotopic compositions of altered oceanic crust: Implications for Si isotope heterogeneity in the mantle. *Chem Geol* 2018; **479**: 1–9.

9. Young ED, Galy A, Nagahara H. Kinetic and equilibrium mass-dependent isotope fractionation laws in nature and their geochemical and cosmochemical significance. *Geochim Cosmochim Acta* 2002; **66**: 1095–1104.

10. Poitrasson F, Zambardi T. An Earth–Moon silicon isotope model to track silicic magma origins. *Geochim Cosmochim Acta* 2015; **167**: 301–312.

11. Savage PS, Georg RB, Armytage RMG *et al.* Silicon isotope homogeneity in the mantle. *Earth Planet Sci Lett* 2010; **295**: 139–146.

12. Pringle EA, Moynier F, Savage PS *et al.* Silicon isotopes reveal recycled altered oceanic crust in the mantle sources of Ocean Island Basalts. *Geochim Cosmochim Acta* 2016; **189**: 282–295.

13. Armytage RMG, Georg RB, Savage PS *et al.* Silicon isotopes in meteorites and planetary core formation. *Geochim Cosmochim Acta* 2011; **75**: 3662–3676.

14. Tang GQ, Su BX, Li QL *et al.* High-mg# olivine, clinopyroxene and orthopyroxene reference materials for in situ oxygen isotope determination. *Geostand Geoanal Res* 2019; **43**: 585–593.

15. Zhang X, Lin CM, Yang SY *et al.* Provenance for the late Quaternary Qiantang River incised-valley fill. *J Palaegeogr* 2018; **20**: 877–892.

16. Zhang WF, Yang Q, Xia XP *et al.* A pb-free sn–bi alloy mount preparation method for secondary ion mass spectrometry (SIMS) analyses. *J Anal At Spectrom* 2024; **39**: 2974–2981.

17. Liu Y, Li XH. New quartz and zircon Si isotopic reference materials for precise and accurate SIMS isotopic microanalysis. *Atom Spectrosc* 2022; **43**: 99–106.

18. Huang C, Wang H, Yu HM *et al.* Further characterization of SA01 and SA02 zircon reference materials for Si and Zr isotopic compositions via femtosecond laser ablation MC-ICP-MS. *J Anal At Spectrom* 2021; **36**: 2192–2201.

19. Villeneuve J, Chaussidon M, Marrocchi Y *et al*. High‐precision in situ silicon isotopic analyses by multi‐collector secondary ion mass spectrometry in olivine and low‐calcium pyroxene. *Rapid Commun Mass Spectrom* 2019; **33**: 1589–1597.

20. Bohrson WA, Spera FJ, Ghiorso MS *et al.* Thermodynamic model for energy-constrained open-system evolution of crustal magma bodies undergoing simultaneous recharge, assimilation and crystallization: the Magma Chamber Simulator. *J Petrol*. 2014; **55**: 1685–1717.

21. Zhou JS, Wang Q, Xing CM *et al.* Crystal growth of clinopyroxene in mafic alkaline magmas. *Earth Planet Sci Lett* 2021; **568**: 117005.

22. Li W, Yang Z, Chiaradia M *et al.* Enrichment nature of ultrapotassic rocks in southern Tibet inherited from their mantle source. *J Petrol* 2021; **62**: egab060.

23. Li W, Yang Z, Chiaradia M *et al.* Redox state of southern Tibetan upper mantle and ultrapotassic magmas. *Geology* 2020; **48**: 733–736.

24. Qin T, Wu F, Wu Z *et al.* First-principles calculations of equilibrium fractionation of O and Si isotopes in quartz, albite, anorthite, and zircon. *Contrib Mineral Petrol* 2016; **171**: 91.

25. Rabin S, Blanchard M, Pinilla C *et al.* Iron and silicon isotope fractionation in silicate melts using first-principles molecular dynamics. *Geochim Cosmochim Acta* 2023; **343**: 212–233.

26. Méheut M, Schauble EA. Silicon isotope fractionation in silicate minerals: Insights from first-principles models of phyllosilicates, albite and pyrope. *Geochim Cosmochim Acta* 2014; **134**: 137–154.

27. Li Y, Wang W, Zhou C *et al.* First-principles calculations of equilibrium silicon isotope fractionation in metamorphic silicate minerals. *Solid Earth Sci* 2019; **4**: 142–149.

28. Rabin S, Blanchard M, Pinilla C *et al.* First-principles calculation of iron and silicon isotope fractionation between Fe-bearing minerals at magmatic temperatures: The importance of second atomic neighbors. *Geochim Cosmochim Acta* 2021; **304**: 101–118.

29. Schmidt MW, Vielzeuf D, Auzanneau E. Melting and dissolution of subducting crust at high pressures: the key role of white mica. *Earth and Planetary Science Letters*. 2004; **228**: 65–84.

30. Kessel R, Ulmer P, Pettke T *et al.* The water–basalt system at 4 to 6 GPa: Phase relations and second critical endpoint in a K-free eclogite at 700 to 1400 °C. *Earth Planet Sci Lett* 2005; **237**: 873–892.

31. Gao M, Foley SF, Xu H *et al.* Mantle metasomatism induced by water-fluxed melting of subducted continental crust at ultrahigh pressures. *Geology* 2023; **51**: 1127–1131.

32. Carter LB, Skora S, Blundy JD *et al.* An experimental study of trace element fluxes from subducted oceanic crust. *J Petrol* 2015; **56**: 1585–1606.

33. Skora S, Blundy JD, Brooker RA *et al.* Hydrous phase relations and trace element partitioning behaviour in calcareous sediments at subduction-zone conditions. *J Petrol* 2015; **56**: 953–980.

34. Martin LAJ, Hermann J. Experimental phase relations in altered oceanic crust: Implications for carbon recycling at subduction zones. *J Petrol* 2018; **59**: 299–320.

35. Sisson TW, Kelemen PB. Near-solidus melts of MORB + 4 wt% H_2_O at 0.8–2.8 GPa applied to issues of subduction magmatism and continent formation. *Contrib Mineral Petrol* 2018; **173**: 70.

36. Skora S, Blundy J. High-pressure hydrous phase relations of radiolarian clay and implications for the involvement of subducted sediment in arc magmatism. *J Petrol* 2010; **51**: 2211–2243.

37. Turner SJ, Langmuir CH. Sediment and ocean crust both melt at subduction zones. *Earth Planet Sci Lett* 2022; **584**: 117424.

38. Turner SJ, Langmuir CH. An alternative to the igneous crust fluid + sediment melt paradigm for arc lava geochemistry. *Sci Adv* 2024; **10**: eadg6482.

39. Ahmad T, Harris N, Bickle M *et al.* Isotopic constraints on the structural relationships between the lesser himalayan series and the high himalayan crystalline series, garhwal himalaya. *Geol Soc Am Bull* 2000; **112**: 467–477.

40. Zeng L, Gao LE, Xie K *et al.* Mid-Eocene high Sr/Y granites in the Northern Himalayan Gneiss Domes: Melting thickened lower continental crust. *Earth Planet Sci Lett* 2011; **303**: 251–266.

41. Workman RK, Hart SR. Major and trace element composition of the depleted MORB mantle (DMM). *Earth Planet Sci Lett* 2005; **231**: 53–72.

42. Liu D, Zhao Z, Zhu DC *et al.* Identifying mantle carbonatite metasomatism through Os–Sr–Mg isotopes in Tibetan ultrapotassic rocks. *Earth Planet Sci Lett* 2015; **430**: 458–469.

43. Miller C, Schuster R, Klotzli U *et al.* Post-collisional potassic and ultrapotassic magmatism in SW Tibet: Geochemical and Sr-Nd-Pb-O isotopic constraints for mantle source characteristics and petrogenesis. *J Petrol* 1999; **40**, 1399–1424.

44. Williams H, Turner S, Kelley S *et al.* Age and composition of dikes in Southern Tibet: New constraints on the timing of east-west extension and its relationship to postcollisional volcanism. *Geology* 2001; **29**: 339.

45. Williams H, Turner S, Pearce J *et al.* Nature of the source regions for post-collisional, potassic magmatism in Southern and Northern Tibet from geochemical variations and inverse trace element modelling. *J Petrol* 2004; **45**, 555–607.

46. Nomade S, Renne PR, Mo X *et al.* Miocene volcanism in the Lhasa block, Tibet: spatial trends and geodynamic implications. *Earth Planet Sci Lett* 2004; **221**: 227–243.

47. Gao Y, Hou Z, Kamber BS *et al.* Lamproitic rocks from a continental collision zone: Evidence for recycling of subducted Tethyan oceanic sediments in the mantle beneath southern Tibet. *J Petrol* 2007; **48**: 729–752.

48. Chen J-L, Xu JF, Wang BD *et al.* Cenozoic Mg-rich potassic rocks in the Tibetan Plateau: Geochemical variations, heterogeneity of subcontinental lithospheric mantle and tectonic implications. *J Asian Earth Sci* 2012; **53**: 115–130.

49. Guo Z, Wilson M, Zhang M *et al.* Post-collisional, K-rich mafic magmatism in south Tibet: constraints on Indian slab-to-wedge transport processes and plateau uplift. *Contrib Mineral Petrol* 2013; **165**: 1311–1340.

50. Liu D, Zhao Z, Zhu DC *et al.* Postcollisional potassic and ultrapotassic rocks in southern Tibet: Mantle and crustal origins in response to India–Asia collision and convergence. *Geochim Cosmochim Acta* 2014; **143**: 207–231.

51. Wang B, Chen J, Xu J *et al.* Geochemical and Sr–Nd–Pb–Os isotopic compositions of Miocene ultrapotassic rocks in southern Tibet: Petrogenesis and implications for the regional tectonic history. *Lithos* 2014; **208**–**209**: 237–250.

52. Huang F, Chen JL, Xu JF *et al.* Os–Nd–Sr isotopes in Miocene ultrapotassic rocks of southern Tibet: Partial melting of a pyroxenite-bearing lithospheric mantle? *Geochim Cosmochim Acta* 2015; **163**: 279–298.

53. Wang R, Richards JP, Zhou LM *et al.* The role of Indian and Tibetan lithosphere in spatial distribution of Cenozoic magmatism and porphyry Cu–Mo deposits in the Gangdese belt, southern Tibet. *Earth-Sci Rev* 2015; **150**: 68–94.

54. Tian S, Yang ZS, Hou ZQ *et al.* Subduction of the Indian lower crust beneath southern Tibet revealed by the post-collisional potassic and ultrapotassic rocks in SW Tibet. *Gondwana Res* 2017; **41**: 29–50.

55. Guo Z, Wilson M. Late Oligocene–early Miocene transformation of postcollisional magmatism in Tibet. *Geology* 2019; **47**: 776–780.

56. Hao LL, Wang Q, Kerr AC *et al.* Contribution of continental subduction to very light B isotope signatures in post-collisional magmas: Evidence from southern Tibetan ultrapotassic rocks. *Earth Planet Sci Lett* 2022; **584**: 117508.

57. Zhang MY, Huang CC, Hao LL *et al.* Light Mo isotopes of post-collisional ultrapotassic rocks in southern Tibet derived from subducted Indian continental crust. *Geochem Geophys Geosyst* 2023; **24**: e2023GC011053.

58. Sun SS, McDonough WF. Chemical and isotopic systematics of oceanic basalts: implications for mantle composition and processes. *Geol Soc Spec Publ* 1989; **42**: 313–345.

59. Chen L, Zheng YF, Zhao ZF *et al.* Continental crust recycling in ancient oceanic subduction zone: Geochemical insights from arc basaltic to andesitic rocks and paleo-trench sediments in southern Tibet. *Lithos* 2022; **414-415**: 106619.

60. Xu JF, Castillo PR. Geochemical and Nd–Pb isotopic characteristics of the Tethyan asthenosphere: implications for the origin of the Indian Ocean mantle domain. *Tectonophysics* 2004; **393**: 9–27.

61. Zhang C, Liu CZ, Wu FY *et al.* Geochemistry and geochronology of mafic rocks from the Luobusa ophiolite, South Tibet. *Lithos* 2016; **245**: 93–108.

62. Zhang SQ, Mahoney JJ, Mo XX *et al.* Evidence for a widespread Tethyan upper mantle with Indian-Ocean-Type isotopic characteristics. *J Petrol* 2005; **46**: 829–858.

63. Inger S, Harris N. Geochemical constraints on leucogranite magmatism in the Langtang Valley, Nepal Himalaya. *J Petrol* 1993; **34**: 345–368.

64. Tian S, Zhao Y, Hou Z *et al.* Lithium isotopic composition and concentration of Himalayan leucogranites and the Indian lower continental crust. *Lithos* 2017; **284**–**285**: 416–428.

65. Liu ZC, Wu FY, Ji WQ *et al.* Monazite record of assimilation and differentiation processes in the petrogenesis of Himalayan leucogranites. *Chem Geol* 2023; **639**: 121700.

66. Cheng Z, Guo Z. Post-collisional ultrapotassic rocks and mantle xenoliths in the Sailipu volcanic field of Lhasa terrane, south Tibet: Petrological and geochemical constraints on mantle source and geodynamic setting. *Gondwana Res* 2017; **46**: 17–42.

67. Lu L, Zhang KJ, Jin X *et al.* Crustal thickening of the central Tibetan Plateau prior to India–Asia collision: Evidence from petrology, geochronology, geochemistry and Sr–Nd–Hf Isotopes of a K-rich charnockite–granite suite in eastern Qiangtang. *J Petrol* 2019; **60**: 827–854.

68. Zhang XZ, Wang Q, Wyman D *et al.* Tibetan Plateau insights into ~1100 °C crustal melting in the Quaternary. *Geology* 2022; **50**: 1432–1437.

69. Zhang XZ, Wang Q, Wyman D *et al.* Tibetan Plateau growth linked to crustal thermal transitions since the Miocene. *Geology* 2022; **50**: 610–614.

70. Malaspina N, Hermann J, Scambelluri M *et al.* Polyphase inclusions in garnet–orthopyroxenite (dabie shan, China) as monitors for metasomatism and fluid-related trace element transfer in subduction zone peridotite. *Earth Planet Sci Lett* 2006; **249**: 173–187.

71. Malaspina N, Hermann J, Scambelluri M. Fluid/mineral interaction in UHP garnet peridotite. *Lithos* 2009; **107**: 38–52.

72. Bali E, Zajacz Z, Kovács I *et al.* A quartz-bearing orthopyroxene-rich websterite xenolith from the pannonian basin, western hungary: Evidence for release of quartz-saturated melts from a subducted slab. *J Petrol* 2008; **49**: 421–439.

73. Rehfeldt T, Foley SF, Jacob DE *et al.* Contrasting types of metasomatism in dunite, wehrlite and websterite xenoliths from kimberley, south africa. *Geochim Cosmochim Acta* 2008; **72**: 5722–5756.

74. Dantas C, Grégoire M, Koester E *et al.* The lherzolite–websterite xenolith suite from northern patagonia (argentina): Evidence of mantle–melt reaction processes. *Lithos* 2009; **107**: 107–120.

75. Rapp RP, Norman MD, Laporte D *et al.* Continent formation in the Archean and chemical evolution of the cratonic lithosphere: Melt-rock reaction experiments at 3-4 GPa and petrogenesis of Archean Mg-diorites (sanukitoids). *J Petrol* 2010; **51**: 1237–1266.

76. Duan X, Sun H, Yang W *et al.* Melt–peridotite interaction in the shallow lithospheric mantle of the north China craton: Evidence from melt inclusions in the quartz-bearing orthopyroxene-rich websterite from hannuoba. *Int Geol Rev* 2014; **56**: 448–472.

77. Ionov DA, Doucet LS, Xu Y *et al.* Reworking of Archean mantle in the NE Siberian craton by carbonatite and silicate melt metasomatism: Evidence from a carbonate-bearing, dunite-to-websterite xenolith suite from the Obnazhennaya kimberlite. *Geochim Cosmochim Acta* 2018; **224**: 132–153.

78. Wang Y, Foley SF, Prelević D. Potassium-rich magmatism from a phlogopite-free source. *Geology* 2017; **45**: 467–470.

79. Förster MW, Buhre S, Xu B *et al.* Two-stage origin of k-enrichment in ultrapotassic magmatism simulated by melting of experimentally metasomatized mantle. *Minerals* 2019; **10**: 41.

80. Ammannati E, Jacob DE, Avanzinelli R *et al.* Low Ni olivine in silica-undersaturated ultrapotassic igneous rocks as evidence for carbonate metasomatism in the mantle. *Earth Planet Sci Lett* 2016; **444**: 64–74.

81. Yang ZF, Li J, Jiang QB *et al.* Using major element logratios to recognize compositional patterns of basalt: implications for source lithological and compositional heterogeneities. *J Geophys Res-Solid Earth* 2019; **124**: 3458–3490.

82. Ruscitto DM, Wallace PJ, Cooper LB *et al.* Global variations in H_2_O/Ce: 2. Relationships to arc magma geochemistry and volatile fluxes. *Geochem Geophys Geosyst* 2012; **13**: 2011GC003887.

83. O’Hara MJ, The bearing of phase equilibria studies in synthetic and natural systems on the origin and evolution of basic and ultrabasic rocks. *Earth-Sci Rev* 1968; **4**: 69–133.

84. Hou Z, Zheng Y, Yang Z *et al.* Contribution of mantle components within juvenile lower-crust to collisional zone porphyry Cu systems in Tibet. *Miner Depos* 2013; **48**: 173–192.

85. Savage PS, Georg RB, Williams HM *et al.* Silicon isotope fractionation during magmatic differentiation. *Geochim Cosmochim Acta* 2011; **75**: 6124–6139.

86. Zeng Z, Sun YF, Tang HY *et al.* Silicon isotope compositions of soil and sediment reference materials determined by MC‐ICP‐MS. *Geostand Geoanal Res* 2022; **46**: 117–127.

87. Zambardi T, Poitrasson F. Precise determination of silicon isotopes in silicate rock reference materials by MC-ICP-MS. *Geostand Geoanal Res* 2011; **35**: 89–99.
